# Paradoxical cancer cell proliferation after FGFR inhibition through decreased p21 signaling in FGFR1-amplified breast cancer cells

**DOI:** 10.1186/s13058-024-01808-7

**Published:** 2024-03-29

**Authors:** Feng Chi, Jason I. Griffiths, Aritro Nath, Andrea H. Bild

**Affiliations:** Department of Medical Oncology and Therapeutics, City of Hope Comprehensive Cancer Institute, 1218 S Fifth Ave, Monrovia, CA 91016 USA

**Keywords:** FGF2, FGFR1, p21, JAK-STAT, Stemness

## Abstract

Fibroblast growth factors (FGFs) control various cellular functions through fibroblast growth factor receptor (FGFR) activation, including proliferation, differentiation, migration, and survival. FGFR amplification in ER + breast cancer patients correlate with poor prognosis, and FGFR inhibitors are currently being tested in clinical trials. By comparing three-dimensional spheroid growth of ER + breast cancer cells with and without FGFR1 amplification, our research discovered that FGF2 treatment can paradoxically decrease proliferation in cells with FGFR1 amplification or overexpression. In contrast, FGF2 treatment in cells without FGFR1 amplification promotes classical FGFR proliferative signaling through the MAPK cascade. The growth inhibitory effect of FGF2 in FGFR1 amplified cells aligned with an increase in p21, a cell cycle inhibitor that hinders the G1 to S phase transition in the cell cycle. Additionally, FGF2 addition in FGFR1 amplified cells activated JAK-STAT signaling and promoted a stem cell-like state. FGF2-induced paradoxical effects were reversed by inhibiting p21 or the JAK-STAT pathway and with pan-FGFR inhibitors. Analysis of patient ER + breast tumor transcriptomes from the TCGA and METABRIC datasets demonstrated a strong positive association between expression of FGF2 and stemness signatures, which was further enhanced in tumors with high FGFR1 expression. Overall, our findings reveal a divergence in FGFR signaling, transitioning from a proliferative to stemness state driven by activation of JAK-STAT signaling and modulation of p21 levels. Activation of these divergent signaling pathways in FGFR amplified cancer cells and paradoxical growth effects highlight a challenge in the use of FGFR inhibitors in cancer treatment.

**Supplementary Information:**

The online version contains supplementary material available at 10.1186/s13058-024-01808-7.

## Background

Breast cancer (BC) is a leading cause of cancer-related deaths among women world-wide [1]. About 3 in 4 breast cancers are estrogen-receptor positive (ER+) [2] and within this group, an estimated 8–15% tumors exhibit FGFR1 amplification [3–6]. This genetic alteration, along with FGFR1 overexpression, is a strong predictor of poor prognosis in ER + breast cancers [7, 8].

FGFR1 is a member of the FGFR family, which comprises four highly conserved transmembrane receptor tyrosine kinases (RTKs), FGFR1-4, and one membrane-associated receptor that lacks the intracellular domain (FGFRL1) [9, 10]. The binding of FGFs to FGFRs activates their extracellular domain, leading to receptor dimerization, the phosphorylation of C-terminal tyrosine, and the activation of downstream signaling pathways, such as PI3K (phosphoinositide 3-kinase) /AKT (Protein kinase B), MAPK (mitogen-activated protein kinase), PLC (phospholipase C)-γ, and JAK (Janus kinase)-STATs (signal transducer and activator of transcription) [11–16]. FGFs were initially discovered to promote the proliferation of fibroblasts and various other cell types, such as keratinocytes, immature osteoblasts, oligodendrocyte progenitors, and endothelial cells [17]. However, these proteins can elicit a wide range of biological responses, including cell proliferation, growth arrest, differentiation, and apoptosis [18]. The FGF family consists of 22 members, which are divided into seven subfamilies [17]. FGF1 (acidic FGF) and FGF2 (basic FGF), which belong to the FGF1 family, can activate all FGFRs [19]. On the other hand, FGF signaling has also been found to cause cell cycle arrest in certain cell types, including PC12 skeletal cells, chondrocytes, and a subset of breast cancer cells [18, 20]. Therefore, understanding how these diverse cellular responses are regulated is a major focus in FGF biology research [18].

In ER + breast cancer, FGFs and FGFR1 amplification promote proliferation by activating MAPK signaling and increase cyclin D1 levels [21, 22]. In contrast, FGF stimulation can also upregulate CDKN1A gene expression, which encodes the p21 protein, in a subset of breast cancer cell lines [20, 23]. The p21 protein acts as an inhibitor of cyclin-dependent kinases (CDKs) by inhibiting the activity of cyclinD-CDK4/6 complexes and the transcription factor E2F, and blocks cell cycle progression during G1 and S phases [24–27]. The expression of CDKN1A gene is regulated by p53, which binds to p53-responsive elements in the CDKN1A promoter to activate its transcription [27], and by CBP (Cyclic AMP response element-binding protein)/p300, which can act as a coactivator with p53 or independently to increase CDKN1A expression by binding to its promoter region [28–30].

FGFR signaling can also activate signal transducers and activators of transcription (STAT) through JAK-STAT pathway, particularly via STAT1 and STAT3 [16]. Studies have shown that STAT1/3 nuclear localization and transcription of downstream target genes includes CDKN1A [31]. STAT1 mediates the growth-inhibitory effect of FGF4 in a breast cancer cell line [23, 27]. By interacting with the transactivation domains of STATs, the co-activator CBP/p300 is recruited, which leads to an enhancement in the transcription rate of target genes [32–35].

The activation of FGFR signaling pathway also enhances stem cell-like characteristics in breast cancer cells [36]. FGF2 can enhance breast cancer mammosphere regeneration, implying its involvement in fostering cancer stem cells (CSCs), a subpopulation exhibiting stem/progenitor properties with the ability for self-renewal [37, 38]. Further, activation of MAPK and JAK/STAT pathways can promote cell survival and the maintenance of stemness in breast CSCs [39].

FGFs and FGFRs are potential targets for cancer treatment [40]. Several receptor-specific and pan-FGFR inhibitors have been developed and tested in clinical trials, either alone or in combination with other drugs [41]; however, they showed little success in ER + breast cancers with FGFR1 amplification [42]. Moreover, the divergent effects of FGF2 on cell cycle and stemness state in breast cancer cells harboring FGFR1 amplification are still unclear. Our study sought to elucidate the dual promotional and inhibitory effects of FGF ligands in ER + breast cancer. By comparing their impact on two subsets of ER + breast cancer cell lines—one with FGFR1 amplification (CAMA1, MDA-MB-134) and the other without (MCF7, T47D)—we confirmed contradictory growth effects and investigated underlying mechanisms. We found that FGF ligands can shift from promoting proliferation to inducing a stem-like state via the JAK-STAT pathway when FGFR1 is amplified concurrent with blocking proliferation. Additionally, our research highlighted potential adverse consequences of using FGFR inhibitors alone in treating ER + breast cancer and provided support for our findings using analyses performed using patient transcriptomic datasets.

## Methods

### Cells and drugs

The drugs listed below were acquired from Selleck chem: FGFR inhibitors PD166866 (specific to FGFR1), Alofanib (targeting FGFR2), H3B-6527 (inhibiting FGFR4), and AZD4547 (effective against FGFR1-3), pan-FGFR inhibitor TAS-120, JAK inhibitors Solcitinib (inhibiting JAK1) and AZD1480 (inhibiting JAK2), UC2288 (p21 inhibitor), STAT inhibitors Fludarabine (inhibiting STAT1),

Stattic (inhibiting STAT3) and BAY2353 (Niclosamide, inhibiting STAT3), SGC-CBP30 (potent CREBBP/EP300 inhibitor), and ulixertinib (ERK1/ERK2 inhibitor). Human EGF protein and FGF ligands including FGF1, FGF2, FGF4, FGF7, FGF9, FGF8a, FG19 and FGF21 were purchased from PeproTech. The breast cancer cell lines CAMA1, MDA-MB-134, MCF7, and T47D were obtained from the American Type Culture Collection (ATCC). The CAMA1 and MCF7 cell lines were grown in DMEM with a 10% FBS and 1% antibiotic-antimycotic solution, while T47D and MDA-MB-134 were cultured in RPMI with 10% FBS and 1% antibiotic-antimycotic solution. Regular testing for mycoplasma contamination was conducted using the commercially available Myco Alert kit from Lonza. All cell lines utilized in this research have undergone authentication by ATCC, and only cells with a low number of passages were employed in experiments to ensure work confidence.

### Statistical analysis

All treatments and analysis were performed in triplicate. All data is presented as the average values of samples, with error bars indicating the standard deviation. We used GraphPad Prism 9.3.1 to generate graphs for experiments except 3D spheroid measurements and performed statistical comparisons of the results using the two-tailed student’s t-test to compare differences between groups as outlined in the figure legends. In all figures depicting qPCR, FACS analysis and mammosphere culture, the DMSO-only treatments (drug concentration = 0) served as controls and were set to a fold of one. A “#” indicates that the FGF2 plus DMSO treatment is significantly higher than the DMSO-only treatment (*p* < 0.05), A “+” indicates that the FGF2 plus DMSO treatment is significantly lower than the DMSO-only treatment (*p* < 0.05), an “*” signifies that the drug plus FGF2 treatment is significantly lower than the DMSO plus FGF2 treatment (*p* < 0.05), and a “^” indicates that the drug plus FGF2 treatment is significantly higher than the DMSO plus FGF2 treatment (*p* < 0.05). In all figures of relative fold change of cell number in + FGF2 vs. -FGF2 spheroids, a “#” indicates that the FGF2 plus drug/DMSO treatment is significantly higher than the drug/DMSO-only treatment inside the group (*p* < 0.05), a “*” indicates that the FGF2 plus drug/DMSO treatment is significantly lower than the drug/DMSO-only treatment inside the group (*p* < 0.05), a “^” indicates that the drug only is significantly higher or lower than the DMSO treatment (*p* < 0.05), and a “+” indicates that the drug plus FGF2 treatment is significantly higher or lower than the DMSO plus FGF2 treatment (*p* < 0.05). In figures of growth rate normalized to inhibitor control (+ FGF2 vs. -FGF2 in each inhibitor dose group) and relative fold change of ratio between cell number in + FGF2 vs. -FGF2 spheroids, the values in DMSO treatment were used as controls. A “#” indicates that the relative ratio of + FGF2/-FGF2 is significantly higher than the DMSO treatment (*p* < 0.05), and an “*” denotes that the relative ratio of + FGF2/-FGF2 is significantly lower than the DMSO treatment (*p* < 0.05). In all figures related cell number estimation from spheroids, one symbol for *p* < 0.05, two symbols for *p* < 0.01, three symbols for *p* < 0.001, and four symbols for *p* < 0.0001.

See additional methods in Supplementary Information.

## Results

### FGF2 inhibits growth by upregulating CDKN1A/p21 levels in FGFR1 amplified ER + breast cancer cells

**Fig. 1:**
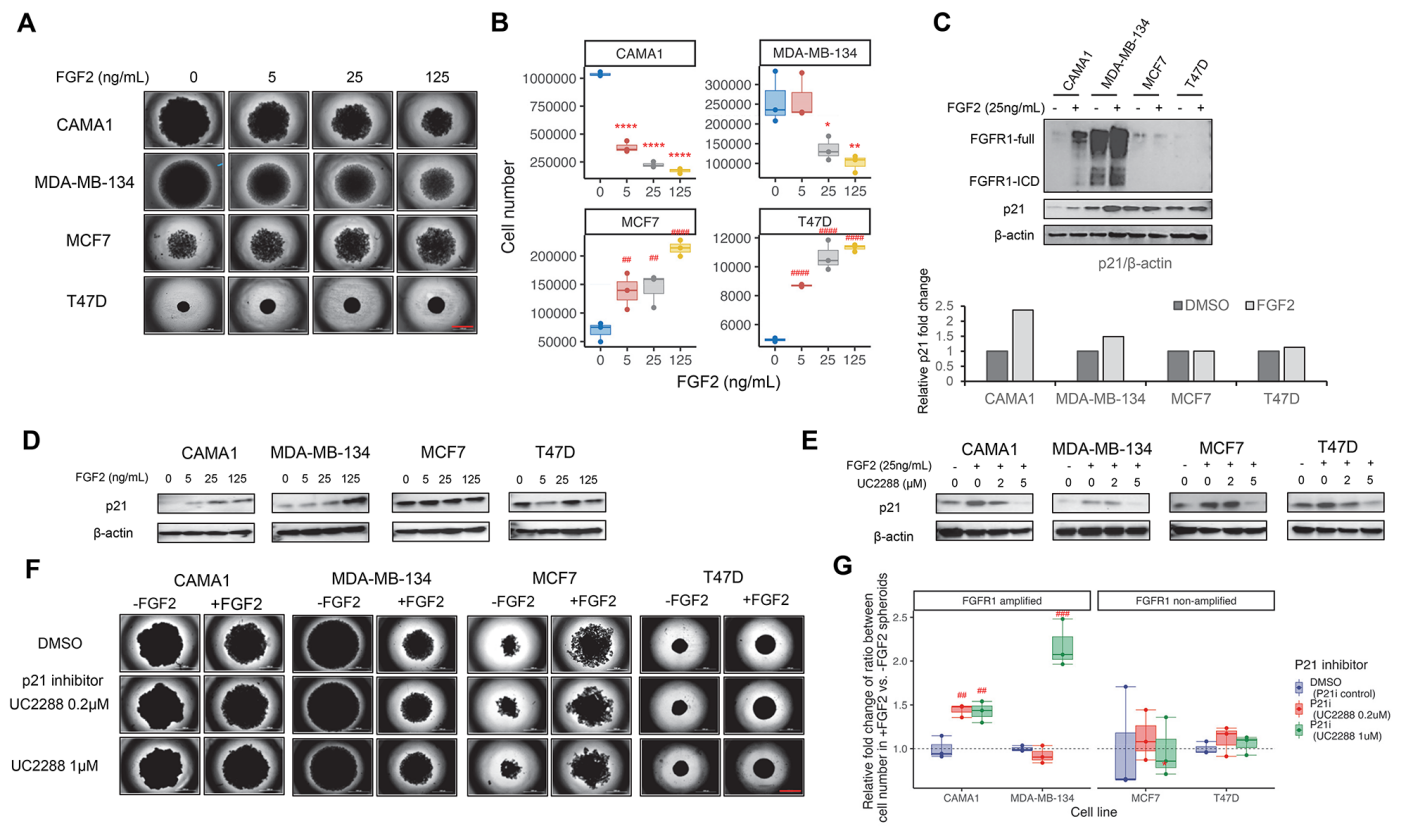
FGF2 induced paradoxical growth effects through p21 modulation in FGFR1 amplified and non-amplified ER + cells. **A**. Spheroid images of FGFR1 amplified cell lines (CAMA1 and MDA-MB-134) and non-amplified cell lines (MCF7 and T47D) show the effects of FGF2 simulation with various doses (5, 25, 125 ng/mL) for 14 days. Bar equals 1000 μm. **B**. Cell number estimation for images of spheroids in Panel **A**, with DMSO treatments serving as controls and set to a fold of one. Asterisks (*) indicate that the FGF2 treatment is significantly lower than the DMSO treatment (*p* < 0.05), while hashtags (#) indicate that the FGF2 treatment is significantly higher than the DMSO treatment (*p* < 0.05). **C**. Comparison of the levels of FGFR1 (full length and ICD) and p21 in ER + cell 3D cultures that are either amplified or non-amplified in FGFR1, with or without FGF2 treatment (25 ng/mL) for 72 h. The relative fold change in p21 for each cell line is determined by the ratio of p21 to β-actin in the FGF2 vs. control. **D**. Immunoblotting shows the expression of p21 in the 3D culture of ER + cells after exposure to various FGF2 dose treatments for 72 h. **E**. Immunoblotting shows p21 protein levels in 3D cell cultures treated with FGF2 (25ng/mL) plus p21 inhibitor UC2288 (2.0 and 5.0 µM) for 72 h. **F**. Spheroid images of all four cell lines show the effects of UC2288 (0.2 and 1.0µM) with or without FGF2 treatment (25 ng/mL) for 14 days. Bar equals 1000 μm. **G**. Relative fold change of ratio between cell number in + FGF2 vs. -FGF2 spheroids in each treatment for the images in Panel **F**, with the ratios in the DMSO treatment serving as controls and set to a fold of one

We studied the effects of FGF2 treatment on proliferation using 3D spheroids of ER + breast cancer cell lines with FGFR1 amplification (CAMA1, MDA-MB-134) and those without (MCF7, T47D). FGF2 showed a dose-dependent reduction in proliferation and size of the 3D spheroids of CAMA1 and MDA-MB-134 cells, while increasing the size of 3D spheroids of MCF7 and T47D cells (Fig. 1A). This outcome was quantified using a parameterized model that integrated both the whole spheroid area and brightfield intensity measurements from each experimental image. This approach allowed the estimation of cell counts for each population during treatment, revealing a significant trend of paradoxical decreased cell abundance in CAMA1 and MDA-MB-134 FGFR1 amplified cells, and a significant increase of cell abundance for MCF7 and T47D cells without FGFR1 amplification with rising concentrations of FGF2, both at the 14-day timepoint (Fig. 1B) and time course throughout the 14-day 3D culture (Fig. S1A). Growth rate of cancer cells estimation exhibited the same trends (Fig. S1B). The confirmation of a paradoxical effect of FGF2 on cancer cell growth was evident in these results, which manifested as a promotion of proliferation in ER + breast cancer cell lines without FGFR1 amplification (MCF7, T47D) and an inhibition of proliferation in those with FGFR1 amplification (CAMA1, MDA-MB-134).

Given that FGFR1 has been identified as an activator of downstream signaling by six subfamilies of FGF family members [43], our subsequent investigation aimed to determine whether other FGF ligands could also elicit the paradoxical growth effect in two groups of cell lines, with or without FGFR1 amplification. For this purpose, we selected one or two FGF ligands from each FGF subfamily, including FGF1, FGF4, FGF7, FGF8a, FGF9, FGF19, and FGF21. Similar to FGF2, rising concentrations of each FGF ligand were incubated with four cell lines, revealing diverse growth effects in spheroid images at 14 days (Fig. S2A) and cell abundance time course analysis over the 14-day 3D culture period (Fig. S2B).

Quantitative analysis was performed by determining the relative fold change in cell number compared to non-treated controls at 14 days. The outcomes varied across different FGF ligands, as depicted in Fig. S2C. For CAMA1, FGF1, FGF4, FGF7, FGF8a, and FGF9 exhibited increased inhibitory effects with rising FGF ligands doses, while FGF19 and FGF21 had no effect within the tested dose range. In MDA-MB-134, FGF1, FGF8a, and FGF9 displayed strong inhibitory effects with increasing doses, while FGF19, and FGF21 exhibited promotional effect, and FGF4 and FGF7 had no effect within the tested dose range. For MCF7, FGF1, FGF4, FGF7, FGF8a, and FGF9 demonstrated promotional effects to varying extents, while FGF21 exhibited inhibitory effects at a high dose, and FGF19 had no effect within the tested dose range. T47D almost shares the same pattern as MCF7 except for no effect with FGF21. In general, paracrine FGF ligands such as FGF1, FGF8a, and FGF9, which rely on heparan sulfate proteoglycan (HSPG) as a coreceptor [43], exhibited comparable behavior to FGF2 and drove paradoxical growth inhibitory responses in FGFR1 amplified cells, while driving growth in cells without FGFR1 amplification, On the other hand, endocrine FGF ligands like FGF19 and FGF21, which utilize Klotho as a coreceptor [43], did not demonstrate a paradoxical growth effect. These findings suggest that the paradoxical growth effect can be induced by paracrine FGF ligands in ER + breast cancer cells.

Western blots confirmed high levels of FGFR1 proteins in the FGFR1-amplified CAMA1 and MDA-MB-134 cells compared to MCF7 and T47D cells. Additionally, FGF2 further increased FGFR1 protein levels in CAMA1 and MDA-MB-134 cells, which correlated with an increase in the level of p21 (Fig. 1C). However, FGF2 did not increase p21 levels in MCF7 and T47D cells. We further observed that p21 expression increased in a dose-dependent manner with FGF2 in the FGFR1-amplified cells, while the non-amplified cell lines do not show this effect (Fig. 1D).

In order to detect a reversal effect of an inhibitor countering the paradoxical growth induced by FGF ligands, we used the following criteria: 1. In the presence of FGF2, the inhibitor augments spheroid size or cell abundance in FGFR1 amplified cells, while reducing these metrics in cells without FGFR1 amplification; 2.The inhibitor notably enhances the relative fold change of ratios between cell numbers in its dose group compared to the DMSO group in FGFR1 amplified cells, but significantly diminishes this ratio in FGFR1 non-amplified cells; 3. Relative to FGF2 treatment alone, the inhibitor combined with FGF2 expedites trajectories for cell abundance over time course in FGFR1 amplified cells while impeding trajectories in FGFR1 non-amplified cells. Alternatively, the emergence of two converging or unified trajectories generated by inhibitor treatment within the same dose group (inhibitor with and without FGF2 addition), when compared to the control group (DMSO and FGF2 alone), could be defined as blocking paradoxical growth effect induced FGF2.

Using UC2288, a p21 expression inhibitor [44], we next evaluated whether the effects of FGF2 on proliferation can be blocked through this signaling pathway. UC2288 decreased p21 levels in all four cell lines (Fig. 1E) and reversed the growth effects of FGF2 by increasing the spheroid sizes of CAMA1 and MDA-MB-134 at the 14-day timepoint (Fig. 1F). However, cells without FGFR1 amplification did not increase in size (Fig. 1F). Relative fold change of cell number was evaluated by comparing FGF2 treated vs. non-treated controls (Fig. S3A) to determine the relative fold change of ratio between cell number in each inhibitor dose group, confirming a significant increase of ratio (+ FGF2 vs. -FGF2) induced by UC2288 compared to the control without drug treatment in CAMA1 and MDA-MB-134, but not in MCF7 and T47D (Fig. 1G). This result suggested inhibition of p21 could reverse the FGF2 effect in FGFR1 amplified cells. Cell number changes in each treatment throughout the 14-day 3D culture time course were quantified in Fig. S3B, and growth rate normalized to inhibitor control showed the same pattern as Fig. 1G (Fig. S3C), confirming the reversal effect of p21 inhibitor. These findings indicated that FGF ligands trigger paradoxical growth in two groups of ER + breast cancer cells, with and without FGFR1 amplification, which corresponds to an increase in the level of p21.

### FGFR1 inhibition causes paradoxical growth in cells with FGFR1 amplification

**Fig. 2:**
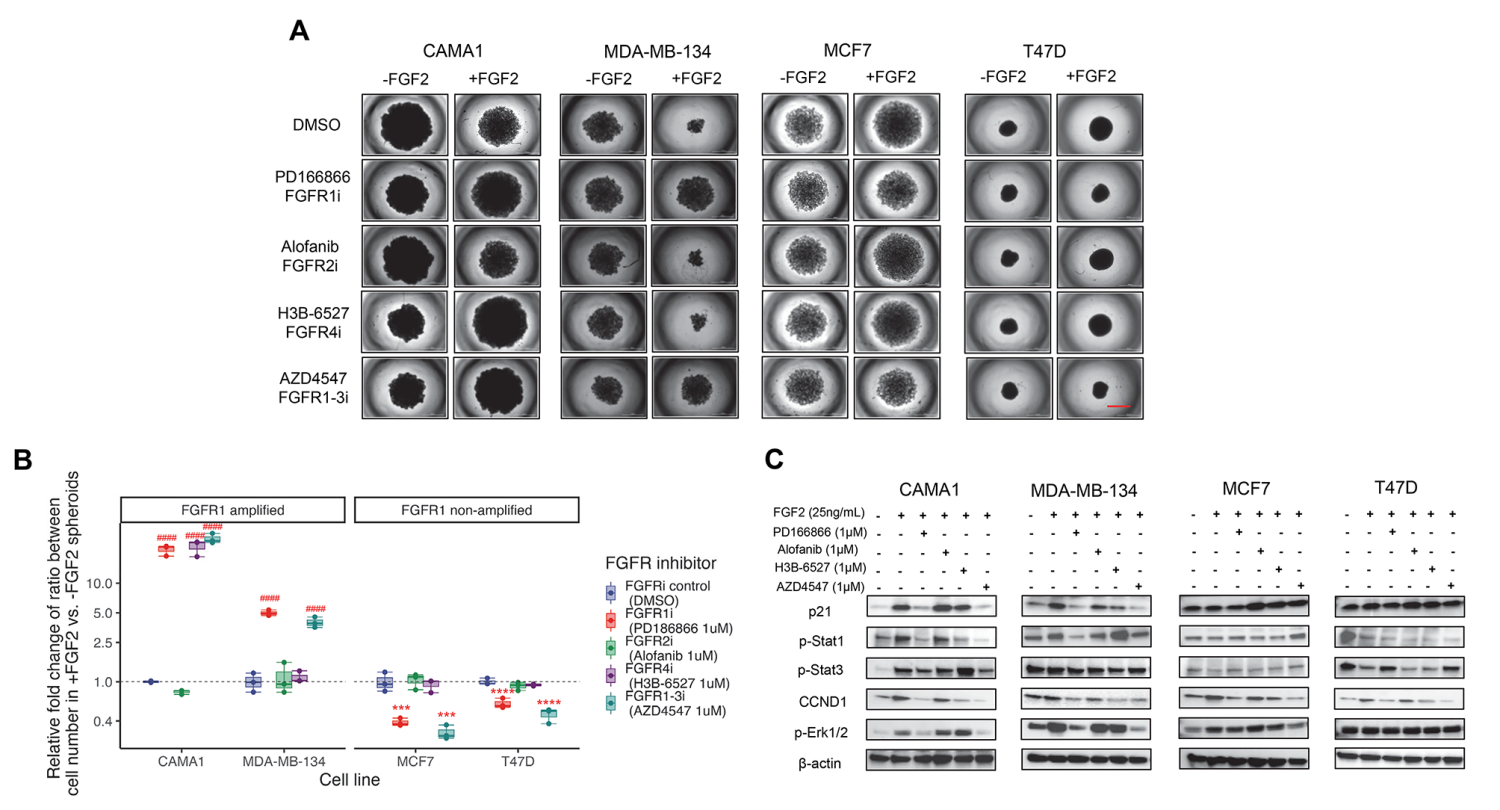
FGF2 caused paradoxical proliferation can be reversed using specific FGFR inhibitors. **A**. Spheroids images formed by FGFR1 amplified cells (CAMA1 and MDA-MB-134) and non-amplified cells (MCF7 and T47D) when incubated with FGFR inhibitors PD166866 (FGFR1i, 1.0µM), Alofanib (FGFR2i, 1.0µM), H3B-6527 (FGFR4i, 1.0µM), and AZD4547 (FGFR1-3i, 1.0µM) in the presence or absence of FGF2 for 14 days. Bar equals 1000 μm. **B**. Relative fold change of ratio between cell number in + FGF2 vs. -FGF2 spheroids for each treatment in Panel **A**. The ratios in the DMSO control group are serving as controls and set as a fold of one. **C**. Immunoblotting shows the levels of p21 and CCND1 expression, as well as the phosphorylation of Stat1/3 and Erk1/2 in ER + cell 3D cultures that were incubated with specific FGFR inhibitors (1.0µM for all inhibitors) and FGF2 (25ng/mL) for 72 h

To characterize the effects of inhibiting FGFR family members on ER + breast cancer cell growth in 3D cultures, we treated the cell lines with or without FGF2 using various inhibitors. These included FGFR1 inhibitor PD166866, FGFR2 inhibitor Alofanib, FGFR4 inhibitor H3B-6527, and FGFR1-3 inhibitor AZD4547. As shown in Fig. 2A, both FGFR1 inhibitor PD166866 and FGFR1-3 inhibitor AZD4547 promoted growth in FGF2 treated CAMA1 and MDA-MB-134 cells when compared to the DMSO with FGF2 treated cells, and inhibited cell proliferation increased by FGF2 in MCF7 and T47D. Cell numbers at 14 days timepoint were quantified by measuring relative fold change through a comparison of FGF2 treated vs. non-treated controls (Fig. S4A) and the relative fold change of ratio in each inhibitor group (Fig. 2B), confirming the reversal effect of PD186866 and AZD4547 on FGF2 induced paradoxical growth in both groups of cell lines. Additionally, a similar increase in cell number was observed in FGF2 treated CAMA1 cells with FGFR4 inhibitor H3B-6527 (Fig. 2A-B, Fig. S4A-B), possibly due to FGFR4 amplification in CAMA1 cells [45]. These results were confirmed by time course analysis of cell abundance change (Fig. S4B), showing accelerated trajectories in CAMA1 and MDA-MB-134 and diminished trajectories in MCF7 and T47D within PD166866 and AZD4547 treatments plus FGF2 compared to other treatments with FGF2 addition. Cancer cell growth rate normalized to inhibitor control (Fig. S4C) exhibited a similar pattern as Fig. 2B, highlighting the paradoxical effect of inhibitors target to FGFR1 in two distinct cell groups. These findings substantiate that the paradoxical growth induced by FGF2 can be reversed by FGFR1 inhibition, which causes posted-treatment paradoxical growth in cells with or without FGFR1 amplification.

Next, we used immunoblotting to examine the protein expression levels of key signal transducers in the 3D cultures with FGF2 treatment and various FGFR inhibitors. First, we confirmed that FGF2 treatment increased p21 levels in both CAMA1 and MDA-MB-134 cells, but not in MCF7 and T47D cells. Next, we found that PD166866 and AZD4547 inhibited the FGF2-increased p21 levels in CAMA1 and MDA-MB-134 (Fig. 2C), which was correlated with the activation of STAT1, but not STAT3. In contrast, p21 expression and STAT1/3 activation were not correlated in MCF7 and T47D cell lines. These results suggest that p21 expression might be regulated through JAK/STAT signaling pathway via STAT1 in FGFR1 amplified cells. In all four cell lines, CCND1 was increased by FGF2, and inhibited by PD166866, H3B-6527 and AZD4547. As a positive regulator of CCND1, Erk1/2 activation was elevated by FGF2 and inhibited by PD166866 and AZD4547 in CAMA1 and MDA-MB-134 cells, which was consistent with p21 level changes (Fig. 2C).

To further investigate the role of the ERK pathway in mediating the effects of FGF2 and FGFR1 amplification on spheroid growth, we used Ulixertinib, a reversible ERK1/ERK2 inhibitor [46]. Immunoblotting analysis confirmed that Ulixertinib inhibited FGF2 promoted ERK activity as indicated by excessive accumulation of p-ERK1/2 [46]. and strongly reduced FGF2 increased CCND1 levels along with a slight decrease in p21 levels, suggesting that Erk1/2 activation promoted CCND1 expression and cell cycle progression (Fig. S5A). Ulixertinib demonstrated a decrease in the relative growth of spheroid sizes when compared to DMSO controls with or without FGF2 treatment, across all cell lines except MCF7 (Fig. S5B). This observation was further validated by assessing the relative fold change in cell number counts at the 14-day timepoint for both FGF2-treated and untreated groups (Fig. S5C), as well as through a time course analysis over a 14-day period (Fig. S5D). Notably, Ulixertinib led to an increase in the relative fold change of cell number ratios within the inhibitor groups for MCF7 and T47D, while inducing a decrease in CAMA1. This contrasted with the pattern observed with the FGFR1 inhibitor (Fig. S5E). These findings suggested that the activation of Erk1/2, and subsequent mediation of cell proliferation through CCND1, is not a pivotal regulator of FGF2-induced paradoxical growth in cells.

### Pan FGFR1-4 inhibitor blocks FGF2 effects in both FGFR1 amplified and non-amplified cells

**Fig. 3:**
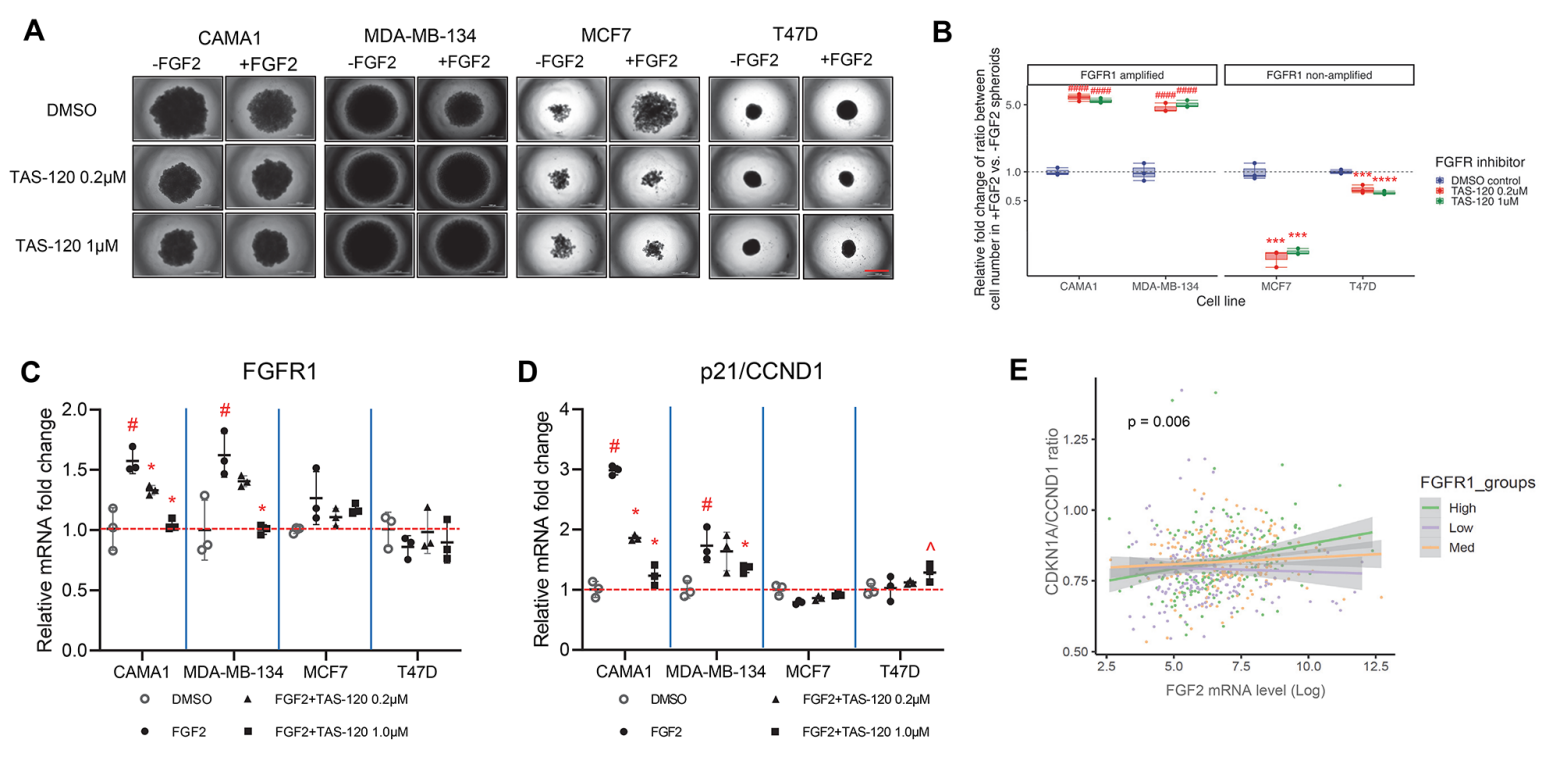
TAS-120 reversed FGF2 induced paradoxical growth effects in FGFR1 amplified and non-amplified ER + BC cells. **(A)** Spheroid images of FGFR1 amplified cells (CAMA1 and MDA-MB-134) and non-amplified cells (MCF7 and T47D) are shown after incubation with different doses (0.2 and 1.0µM) of TAS-120 and FGF2 (25 ng/mL) for 14 days. Bar equals 1000 μm. **(B)** Relative fold change of ratio between cell number in + FGF2 vs. -FGF2 spheroids in Panel **A** are depicted, with the DMSO control group serving as controls and set to a fold of one. **C-D.** Relative mRNA expression level of FGFR1 (**C**) and ratio of p21/CCND1 (**D**) in ER + cell 3D cultures incubated with different doses (0.2 and 1.0 µM) of TAS-120 plus FGF2 (25 ng/mL) for 72 h. The mRNA expression was normalized to RPLP0 and the control treatments, using DMSO, are set to a fold of one. **E.** Plots that depict the relationship between the expression levels of FGF2 (X-axis) and the CDKN1A/CCND1 ratio (Y-axis) in 601 ER + breast cancer patients from TCGA. The three linear fit curves and 95% C.I. (grey shaded area) demonstrate the interaction effects between FGF2 and FGFR1 expression, where FGFR1 expression levels are grouped into tertiles (high *n* = 206; low *n* = 199; med *n* = 196)

To investigate the role of FGFR1 in the regulation of p21 levels in ER + breast cancer cell lines, we used the pan-FGFR inhibitor TAS-120 (irreversible FGFR1-4 inhibitor) to block the effects of FGF2. TAS-120 reduced the spheroid sizes of MCF7 and T47D that increased by FGF2 while it reversed the decrease in spheroid sizes of MDA-MB-134 caused by FGF2 (Fig. 3A). Relative fold change of cell number indicated although TAS-120 further reduced cell number of CAMA1 compared to FGF2 treatment only, the cell abundance with or without FGF2 addition under the same TAS-120 dose treatment were relatively close without significant difference, and such situation was present in all four cell lines (Fig. S6A). The relative fold change of ratio between cell numbers within the same inhibitor dose group revealed a significant increase in CAMA1 and MDA-MB-134, coupled with a substantial decrease in MCF7 and T47D (Fig. 3B). The time course analysis of cell number counts revealed that TAS-120 completely abolished the impact of FGF2 in both cell groups (Fig. S6B). The cancer cell growth rate, when normalized, corroborated a consistent pattern with the observations in Fig. 3B (Fig. S6C). These results indicated that by blocking all FGFRs, TAS-120 could inhibit FGF2 effects in both FGFR1 amplified and non-amplified cells.

Next, we evaluated the transcription levels of CDKN1A vs. CCND1 using RT-qPCR (primer sequences are provided in Table S1). FGF2 was found to upregulate FGFR1 and CDKN1A mRNA levels in the CAMA1 and MDA-MB-134 cells, but not in the non-FGFR1 amplified MCF7 and T47D cells, consistent with the Western blot results in Fig. 1C, while TAS-120 inhibition decreased mRNA levels of both FGFR1 and p21 (Fig. 3C, Fig. S6D). In contrast, CCND1 mRNA levels were increased by FGF2 treatment in three of four cell lines (except MDA-MB-134) (Fig. S6E). Prior studies indicated the ratio of CDKN1A/CCND1 is important for measuring cell growth modulation [47]. We next calculated the ratio of CDKN1A vs. CCND1 to evaluate the balance of these anti- and pro-growth signals, respectively, in each cell line and treatment. The results showed CAMA1 and MDA-MB-134 exhibited a relatively higher level of growth inhibitory effects after FGF2 treatment, which is decreased with the addition of TAS-120, while MCF7 and T47D exhibited a relatively low difference compared to control untreated (Fig. 3D), The alterations in the CDKN1A vs. CCND1 ratio remained consistent with the paradoxical growth effects induced by FGF2 and TAS-120, as confirmed through 3D culture, supporting its potential role as an indicator for discerning the anti- or pro-growth effects in individual samples under FGF ligand stimulation.

We next analyzed the associations between FGF2 and FGFR1 gene expression levels and the expression of CDKN1A or CCND1 in the tumor transcriptomes of 601 ER + breast cancer patients from the Cancer Genome Atlas (TCGA). Using generalized linear models, we found that increasing FGF2 expression was not significantly associated with CDKN1A expression (*p* = 0.09) (Fig. S7A). However, higher FGF2 expression was associated with reduced CCND1 levels (*p* = 0.004) (Fig. S7B) and a significant increase in the CDKN1A/CCND1 ratio (*p* = 0.004) (Fig. S7C). On the other hand, FGFR1 expression levels, partly driven by FGFR1 amplifications, were associated with a significant increase in the expression levels of both CDKN1A (*p* = 8.6 × 10^− 11^) and CCND1 (4.6 × 10^− 6^) (Fig. S7D-E). Consequently, elevated FGFR1 expression did not result in a significant change in the CDKN1A/CCND1 ratios (*p* = 0.36) (Fig. S7F). Further, the associations observed between FGFR1 and the CDKN1A, CCND1 or CDKN1A/CCND1 ratios were not observed in samples segregated by TP53 mutations (Fig. S7G-I), suggesting these patterns were not TP53-dependent.

We further explored interactions between FGF2 and FGFR1 expression in the generalized linear models and their impact on CDKN1A, CCND1 and CDKN1/CCND1 ratio. We found no significant interaction effects between FGF2 and FGFR1 in the CDKN1A model (*p* = 0.172) (Fig. S8A). However, we observed a significant negative interaction effect in the CCND1 model (*p* = 0.014), suggesting that at high FGFR1 expression levels, FGF2 had a greater negative association with CCND1 expression (Fig. S8B). There was a significant positive interaction effect in the CDKN1A:CCND1 model (*p* = 0.006), suggesting that at high FGFR1 expression levels, FGF2 was associated with an elevated CDKN1A/CCND1 ratio (Fig. 3E). These results suggested that a high level of FGF2 shifts ER + breast cancer cells from progression to regression by increasing the CDKN1A/CCND1 ratios in cells with elevated FGFR1.

### FGF2 blocks G1 to S phase transition in FGFR1 amplified cells while FGFR1 inhibitors reverse this effect

**Fig. 4:**
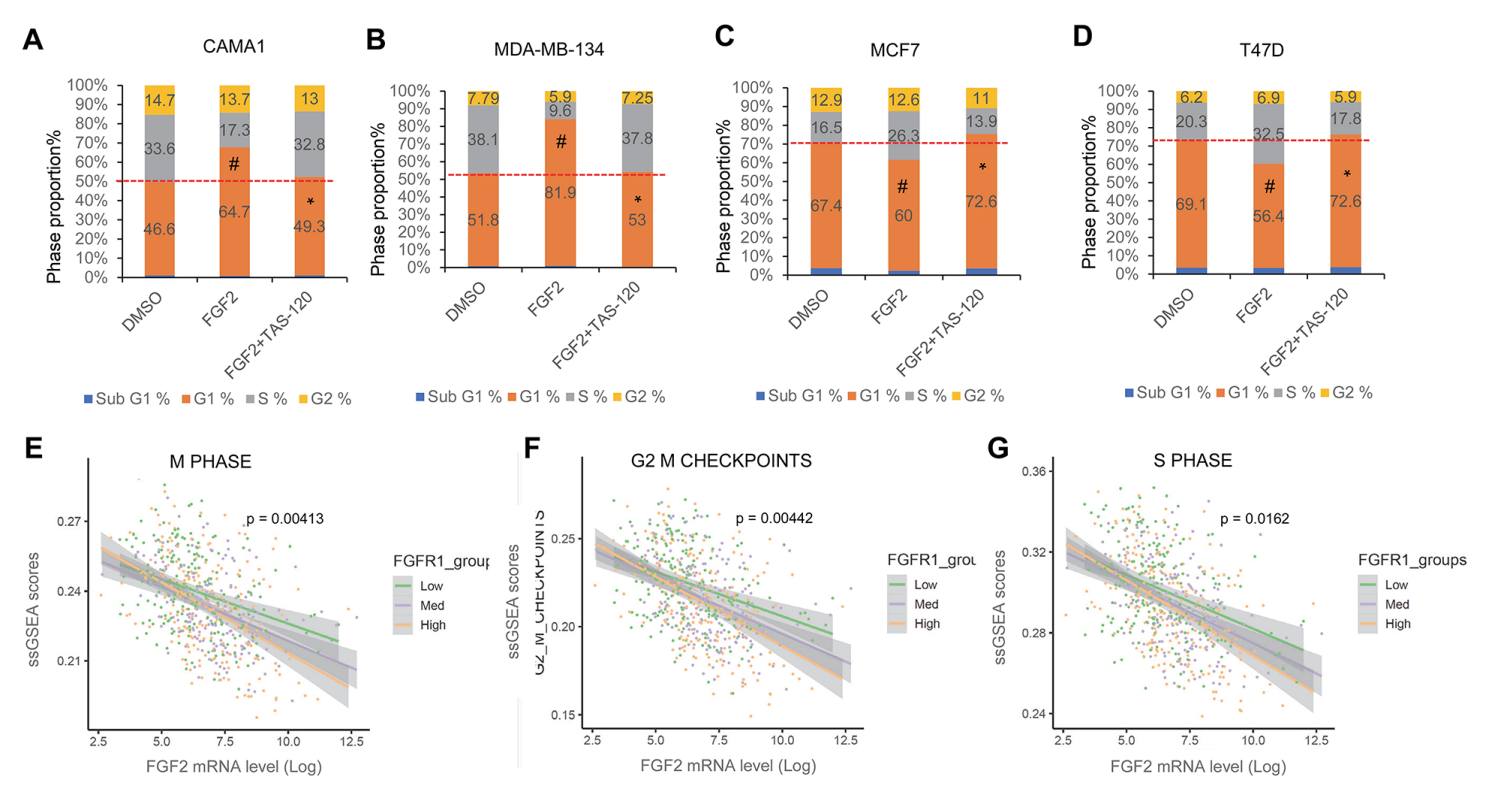
FGF2 lead to opposite effects on cell cycle in FGFR1 amplified and non-amplified ER + cells. **A-D**. The proportion of cells in each phase of the cell cycle was evaluated through PI staining-based flow cytometry analysis in FGFR1 amplified cell lines CAMA1 and MDA-MB-134 (**A** and **B**) and non-amplified cell lines MCF7 and T47D (**C** and **D**). The cells were collected from 2D cultures after being exposed to FGF2 (25ng/ml) with or without TAS-120 (1.0 µM) for 24 hours. The symbol “#’ indicates that the G1 phase in FGF2-treated cells is significantly higher compared to DMSO-treated cells (*p* < 0.05). The symbol “*’ shows that the G1 phase in FGF2 plus TAS-120 treated cells is significantly lower compared to FGF2-treated cells (p < 0.05). **E-G.** TCGA ER + breast cancer patients data analysis for interactions in the generalized linear model between FGF2 (X-axes):FGFR1 expression and their impact on GSEA pathways related to M phase (**C**), G2/M checkpoints (**F**) and S phase (**G**) (Y-axes, n = 601). The three linear fit curves and 95% C.I. (grey shaded area) are shown to indicate the interaction effects between FGF2 and FGFR1 expression, where FGFR1 expression levels were grouped by tertiles (high n = 206; low n = 199; med n = 196)

To show that p21 is a crucial regulator mediating the effects of FGF2 on cell proliferation in FGFR1 amplified cancer cells, we used propidium iodide (PI)-based cell cycle analysis with flow cytometry (Fig. 4A-B; Table 1). Our results indicated that FGF2 caused G1 to S phase arrest in CAMA1 and MDA-MB-134 cells by increasing the proportion of cells in G1 phase (from 46.6 to 64.7% in CAMA1 and from 51.8 to 81.9% in MDA-MB-134) and decreasing the proportion in S phase (from 33.6 to 17.3% in CAMA1 and from 38.1 to 9.6% in MDA-MB-134). The addition of TAS-120 eliminated these effects. On the other hand, FGF2 promoted cell cycle progression in MCF7 and T47D cells by decreasing the proportion of cells in G1 phase (from 67.4 to 60% in MCF7 and from 69.1 to 56.4% in T47D) and increased the proportion of cells in S phase (from 16.5 to 26.3% in MCF7 and from 20.3 to 32.5% inT47D), but TAS-120 blocks this effect (Fig. 4C-D; Table 1). Detailed and merged FACS plots of three treatments confirmed that TAS-120 completely reversed FGF2-driven cell cycle arrest in CAMA1 and MDA-MB-134 (Fig. S9A-B) and cell cycle progression in MCF7 and T47D (Fig. S9C-D). Our data confirmed these divergent effects of FGF2 on cell cycle in these two groups of cell lines and supports the key role of p21 in FGF2-FGFR1 regulated pathways that inhibit proliferation.

**Table 1:** Cell cycle phase proportions in ER + BC cells with or without TAS-120 treatment

Cell lines	Treatment	Sub G1%	G1%	S%	G2%
CAMA1	DMSO	1.1 ± 0.3	46.6 ± 2.8	33.5 ± 1.0	14.7 ± 2.2
	FGF2	0.8 ± 0.1	64.7 ± 1.6	17.3 ± 0.6	13.7 ± 0.6
	FGF2 + TAS-120	1.1 ± 0.4	49.2 ± 3.9	32.8 ± 3.5	13.0 ± 0.9
MDA-MB-134	DMSO	0.9 ± 0.5	51.7 ± 2.7	38.1 ± 2.3	7.8 ± 0.7
	FGF2	1.0 ± 0.5	81.8 ± 3.1	9.6 ± 1.2	5.9 ± 0.2
	FGF2 + TAS-120	0.3 ± 0.1	53.0 ± 1.1	37.8 ± 0.4	7.2 ± 0.4
MCF7	DMSO	3.8 ± 0.6	67.4 ± 2.9	16.5 ± 0.5	12,9 ± 1.8
	FGF2	2.4 ± 0.2	60.0 ± 1.7	26.3 ± 2.3	12.5 ± 3.9
	FGF2 + TAS-120	3.6 ± 0.8	72.6 ± 0.4	13.9 ± 1.0	10.9 ± 1.2
T47D	DMSO	3.5 ± 0.2	69.1 ± 1.2	20.3 ± 0.7	6.2 ± 0.5
	FGF2	3.5 ± 0.7	56.4 ± 2.2	32.5 ± 1.4	6.9 ± 0.7
	FGF2 + TAS-120	3.8 ± 0.1	72,6 ± 1.4	17.8 ± 1.1	5.9 ± 1.3

The cells were collected from 2D cultures after being exposed to FGF2 (25ng/ml) with or without TAS-120 (1.0 µM) for 24 h, followed by PI staining-based flow cytometry analysis. The proportions of each phase were determined by Flowjo V10.3

The results from the analysis of gene expression data from TCGA ER + breast cancer patients using generalized linear models based on also showed a correlation between FGF2, FGFR1 and cell cycle states. Our analysis indicated that elevated FGF2 levels were associated with a decrease in the M phase (Fig. 4E), G2/M checkpoints (Fig. 4F), and S phase (Fig. 4G) signature scores. This correlation was particularly pronounced at high levels of FGFR1, as indicated by the FGF2:FGFR1 interaction model. We confirmed these results using the ER + breast cancer transcriptomes from the METABRIC (Molecular Taxonomy of Breast Cancer International Consortium) dataset. These analyses yielded similar results, indicating that elevated FGF2 and FGFR1 levels strongly reduced cell cycle-related pathway signature scores including M phase (Fig. S9E), G2/M checkpoints (Fig. S9F), and S phase (Fig. S9G). These results suggest that high levels of FGFR1 could inhibit mitosis upon FGF2 stimulation, which supports our experimental cell cycle analysis results for two groups of ER + breast cancer cells.

### FGFR1 overexpression mimics FGFR1 amplification and upregulates p21 to inhibit cell cycle progression

**Fig. 5:**
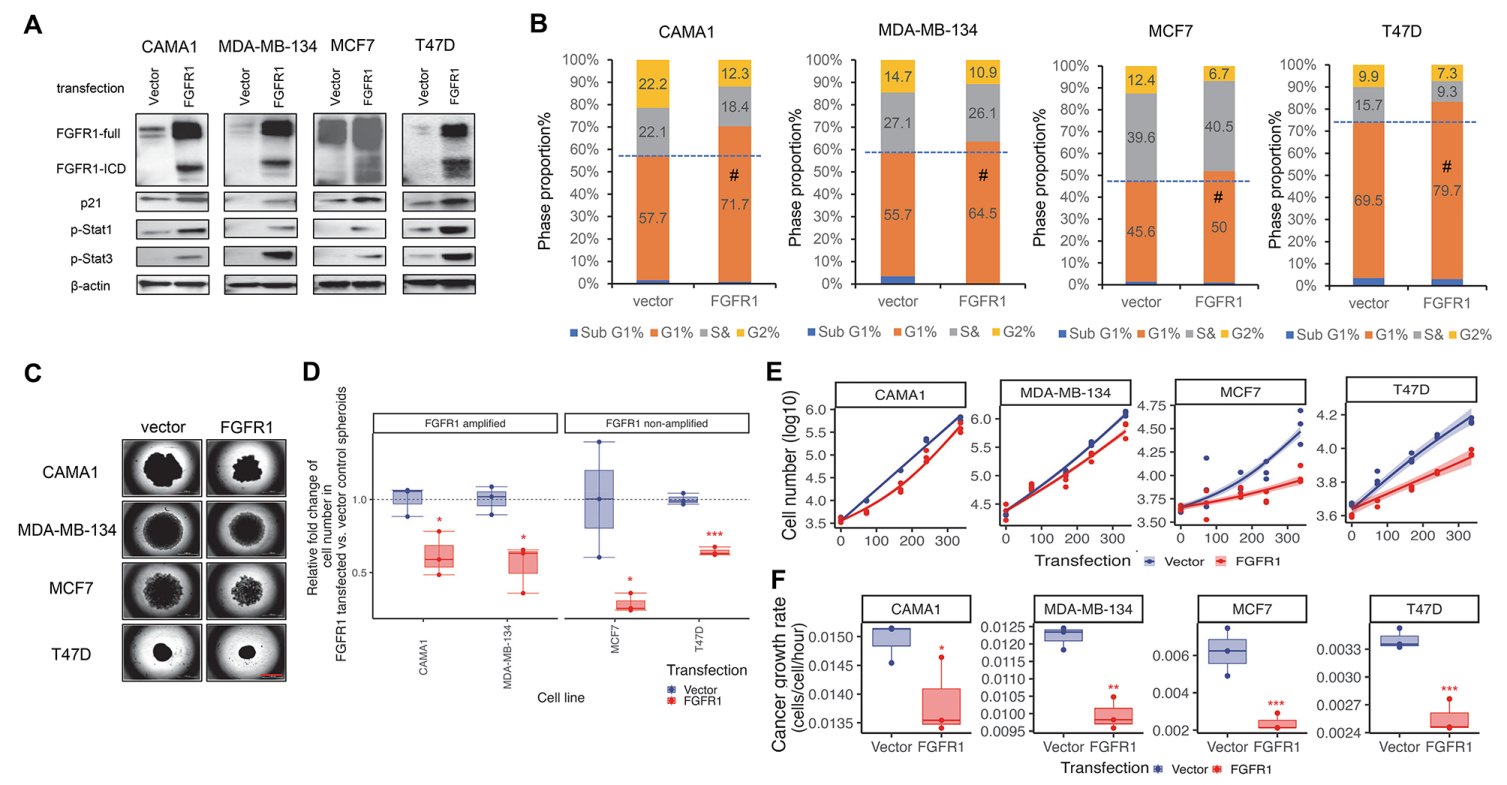
FGFR1 overexpression upregulated p21 to inhibit cell cycle progression. **A**. Immunoblotting shows the levels of FGFR1, p21, and stat1/stat3 activation in 2D cultures of FGFR1 amplified cells (CAMA1 and MDA-MB-134) and non-amplified cells (MCF7 and T47D) after transfection with pCMV-XL6-FGFR1 plasmid or an empty pCMV-XL6 vector (as controls). **B**. Cell cycle phase proportions were determined by PI staining-based cell cycle FACS analysis in all four cell lines cells with FGFR1 and empty vector transfection in 2D culture. A ‘#’ symbol indicates G1 phase in FGFR1 transfected cells significantly higher than in empty vector transfected cells; **C**. 3D spheroid images are shown for four cell lines with FGFR1 and empty vector transfection after 14 days 3D culture. Bar equals 1000 µm. **D**. Relative fold change of cell number in FGFR1 transfected vs. vector control spheroids for images in (**C**). The cells transfected with empty vector are used as controls and set as fold one. **E**. Time course of cell number change over 14 days culture. for FGFR1 and vector control transfected cell lines. **D** and **F**, a ‘*’ symbol indicates the cells transfected with FGFR1 significantly lower than the cells transfected with empty vector (p < 0.05). **F**. cancer cell growth rate for cells transfected with FGFR1 and vector control. For **D** and **F**, a ‘*’ symbol indicates the cells transfected with FGFR1 significantly lower than the cells transfected with empty vector (*p* < 0.05)

To further test the link between amplified FGFR1 and p21 regulation, we overexpressed FGFR1 in CAMA1, MDA-MB-134, MCF7, and T47D cells using an FGFR1 vector transfection. The cells were then analyzed using immunoblotting, cell cycle analysis, and 3D spheroid growth. Both the full-length and intracellular domain (ICD) of FGFR1 were overexpressed in all four cell lines compared to the empty vector transfected cells, resulting in p21 upregulation and STAT1/3 activation (Fig. 5A). The cell lines with FGFR1 transfection showed a significant increase in the G1 phase of the cell cycle compared to the empty vector transfected cells, with G1 phase increasing from 51.7 to 71.7% in CAMA1 cells, 55.7 to 64.5% in MDA-MB-134 cells, 45.6 to 50.0% in MCF7 cells, and from 61.7 to 69.8% in T47D cells (Fig. 5B; Table 2). FACS plots of cells with FGFR1 and empty vector transfection confirmed that FGFR1 overexpression resulted in cell cycle arrest in all four cell lines (Fig. S10A-D). These results were additionally validated through spheroid images of transfected cells at 14 days timepoint (Fig. 5C) and relative fold change of cell number in FGFR1 transfected vs. vector controls for Fig. 5C (Fig. 5D). The trajectory of the time course analysis for cell number counts (Fig. 5E) and the estimation of cancer cell growth rate (Fig. 5F) showed a decrease in growth attributed to FGFR1 overexpression when compared to the vector control, indicating that FGFR1 overexpression can inhibit breast cancer cell proliferation by upregulating p21.

**Table 2:** Cell cycle phase proportions in ER + BC cells with or without FGFR1 overexpression

Cell lines	Transfection	Sub G1%	G1%	S%	G2%
CAMA1	vector	1.7 ± 0.8	57.7 ± 2.0	22.1 ± 1.4	22.2 ± 1.6
	FGFR1	1.0 ± 1.2	71.6 ± 2.5	18.4 ± 2.5	12.2 ± 1.4
MDA-MB-134	vector	0.7 ± 0.5	55.2 ± 0.5	31.2 ± 0.4	15.8 ± 1.2
	FGFR1	0.5 ± 0.4	59 ± 1.6	31 ± 0.8	9.8 ± 1.1
MCF7	vector	1.4 ± 0.7	45.6 ± 1.0	39.6 ± 2.3	12.3 ± 4.8
	FGFR1	1.0 ± 0.3	50 ± 1.0	40.5 ± 2.9	6.7 ± 3.5
T47D	vector	6.5 ± 1.3	61.7 ± 2.0	23.7 ± 1.9	6.5 ± 1.1
	FGFR1	6.7 ± 0.6	69.8 ± 1.3	18.1 ± 1.3	6.2 ± 0.4

The cells were transfected with FGFR1 or empty vectors and collected after 48 h, followed by PI staining-based flow cytometry analysis. The proportions of each phase were determined by Flowjo V10.3

JAK-STAT and CBP pathways regulate growth inhibition by p21 in FGFR1 amplified cells.

**Fig. 6:**
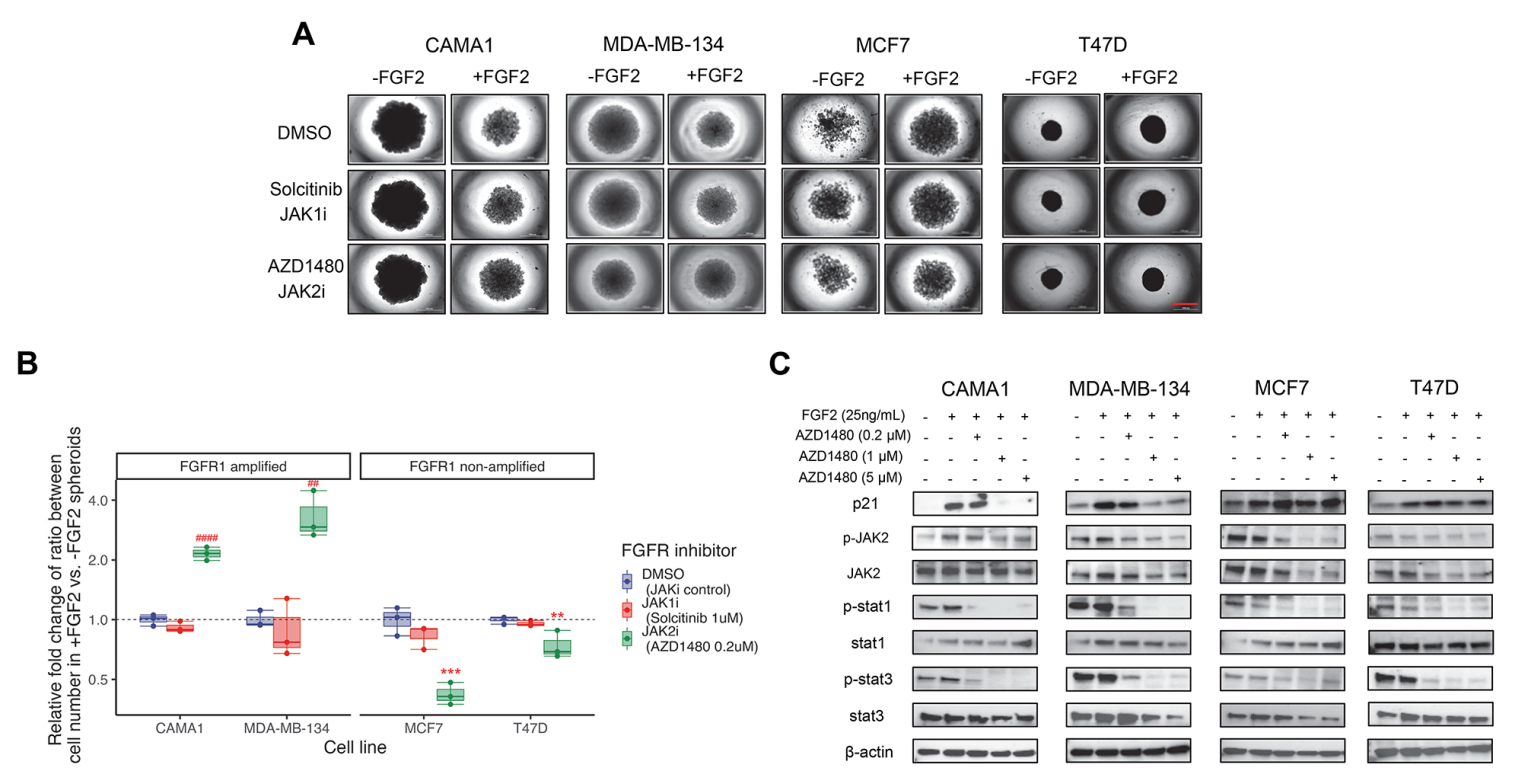
The JAK2 inhibitor AZD1480 reversed the paradoxical proliferation caused by FGF2. **A**. Spheroid images show the effects of the JAK1 inhibitor Solcitinib (1.0 µM) and the JAK2 inhibitor AZD1480 (0.2 µM) on FGFR1 amplified cells (CAMA1 and MDA-MB-134) and non-amplified cells (MCF7 and T47D) with and without FGF2 treatment (25 ng/mL) for 14 days. Bar equals 1000 μm. **B**. Relative fold change of ratio between cell number in + FGF2 vs. -FGF2 spheroids is calculated for each treatment. The ratios in DMSO treatment are used as controls and are set to a fold of one. **C**. Immunoblotting shows the effect of specific FGFR inhibitors (1.0 µM for all FGFR inhibitors) on p21 expression levels, JAK2 and Stat1/3 activation in ER + cell 3D cultures after 72 h of treatment with FGF2 (25 ng/mL)

To determine if the effects of p21 in FGFR1 amplified cells are regulated by the JAK-STAT pathway, we treated FGFR1 amplified and non-amplified cell lines with or without FGF2 in 3D cultures with Solcitinib, a JAK1 inhibitor and AZD1480, a JAK2 inhibitor. Imaging of spheroids on day 14 revealed that the JAK2 inhibitor AZD1480 countered the FGF2 induced shrinkage of 3D spheroids in FGFR1 amplified cells (Fig. 6A). Inhibition of JAK2 also increased the relative growth of spheroids that were reduced by FGF2 vs. controls without FGF2 in CAMA1 and MDA-MB-134 cells, while reducing the relative growth of spheroids increased by FGF2 treatment vs. controls without FGF2 in MCF7 and T47D cells (Fig. 6A). These results were confirmed by relative fold change of cell number by comparing FGF2 treated vs. non-treated controls (Fig. S11A) and the relative fold change of ratio between cell number in each inhibitor dose group (Fig. 6B). JAK1 inhibitor Solcitinib had no impact on 3D spheroid growth compared to controls in all four cell lines, suggesting that regulation of p21 in FGFR1 amplified cells may occur through JAK2, but not JAK1. The time course analysis of cell number (Fig. S11B) and cancer cell growth rate normalized to inhibitor control (Fig. S11C) provided additional confirmation of the ability of the JAK2 inhibitor AZD1480 to counteract the paradoxical growth induced by FGF2 in cells with or without amplified FGFR1, In line with these results, immunoblotting indicated that AZD1480 inhibited the activation of JAK2 and STAT1/3 in both groups of cell lines, but only strongly reduced the level of p21 only in CAMA1 and MDA-MB-134 with a dose-dependent manner (Fig. 6C), confirming the role of the JAK-STAT pathway in the regulation of p21 in FGFR1 amplified cells.

STAT1 has been recognized as regulator of p21 expression through the FGFR pathway in breast cancer [23]. In addition, STAT3 has also been identified as a regulator of CDKN1A transcription in various cancer cells [48–50]. Therefore, we investigated the roles of STAT1 and STAT3 in p21 regulation in FGF2-induced growth effects associated with FGFR1 amplification. In our study, we treated the four cell lines with Fludarabine (a STAT1 inhibitor), Stattic (a STAT3 inhibitor), and BAY2353 (another STAT3 inhibitor). Immunoblotting results indicated that Fludarabine inhibited FGF2-activated STAT1 in all four cell lines, leading to a decrease in p21 when compared to FGF2 treatment alone. Compared to STAT1 inhibitor, BAY2353 blocked FGF2-activated STAT3 and p21 levels in CAMA1, MDA-MB-134 and T47D, while Stattic could only inhibit p21 in MDA-MB-134 and T47D, but not in CAMA1 (Fig. S12A). We then tested Fludarabine and BAY2353 for 3D cell culture with FGF2 addition. Our analysis of spheroid images obtained on day 14 (Fig. S12B) and the relative fold change of cell numbers (-FGF2 vs. +FGF2) (Fig. S12C) revealed that both STAT inhibitors reduced spheroid sizes and cell abundance to a relatively similar ratio between treatments with or without FGF2 addition in both CAMA1 and MDA-MB-134. However, only Fludarabine, and not BAY2353, exhibited a similar effect in MCF7 and T47D. The growth reversal effect of both Fludarabine and BAY2353 was further tested in Fig. S12C, demonstrating a significantly increased relative fold change of the ratio between cell numbers in each inhibitor group compared to the DMSO group in both CAMA1 and MDA-MB-134 (Fig. S12D). Additionally, analysis of the trajectory of cell numbers over the time course (Fig. S12E) and the cell growth rate normalized to the inhibitor control (Fig. S12F) confirmed that both inhibitors resulted in a unified trajectory with or without FGF2 addition and increased normalized cell growth rate. These results demonstrated the ability of both Fludarabine and BAY2353 to reverse the FGF2-induced growth effect in cell lines with amplified FGFR1, but not in cells without FGFR1 amplification. Moreover, these findings indicated that both STAT1 and STAT3 can modulate p21 regulation in ER + breast cancer cells and contribute to FGF2 induced paradoxical growth effects.

We next examined the role of CBP/p300 in regulating FGF2 effects on cell proliferation, given that CBP/p300 interacts with STATs and increases the transcription rate of target genes as a co-activator [32–35]. SGC-CBP30, a potent and selective inhibitor of CBP/p300, reversed the relative growth of spheroids following FGF2 treatment in CAMA1 and MDA-Mb-134 cells (Fig. S13A). This was confirmed by the relative fold change of cell number through a comparison of FGF2 treated vs. non-treated controls (Fig. S13B) and the relative fold change of ratio between cell numbers in each inhibitor dose group (Fig. S13C). Time course analysis of cell number (Fig. S13D) and growth rate normalized to inhibitor control (Fig. S13E) showed the reversal effect of SGC-CBP30 in FGFR1 amplified cells with FGF2 addition, Also, SGC-CBP30 decreased p21 levels in all four cell lines in a dose-dependent manner (Fig. S13F), suggesting that CBP/p300 contributes to regulation of p21 expression in FGFR1-amplified cells.

### FGF2 and FGFR1 amplification promote cancer stemness through the JAK-STAT pathway

**Fig. 7:**
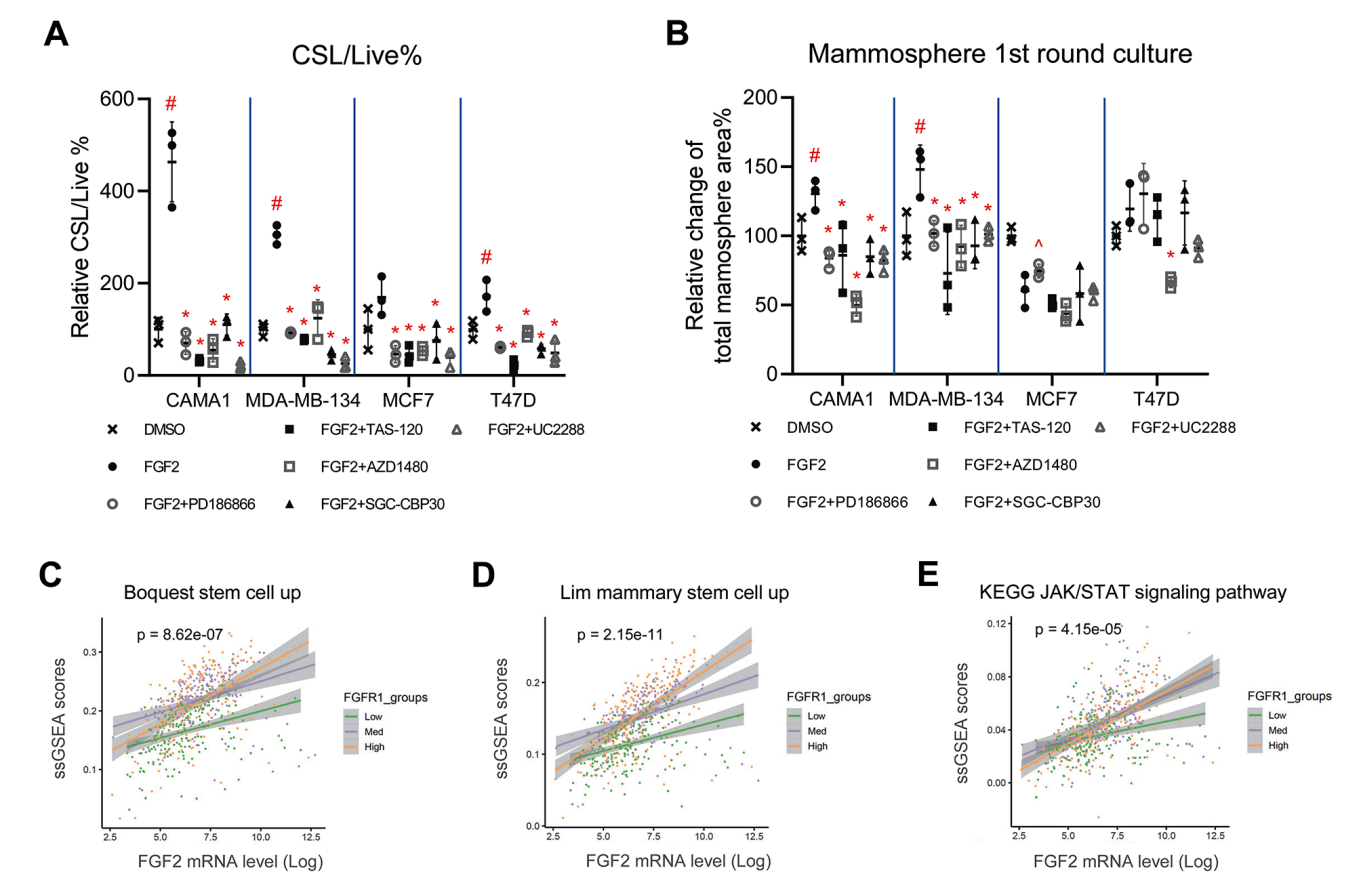
Amplified FGFR1 promotes FGF2 induced cancer stemness through JAK-STAT pathway. **A-B.** Relative percentage changes of CSL cells/live cells (**A**) and relative percentage change of total mammosphere area for the 1st round culture (**B**) are shown in four ER + cell 3D cultures incubated with FGF2 (25ng/mL) plus PD166866 (1.0µM), TAS-120 (1.0µM), AZD1480 (0.2µM), SGC-CBP30 (10µM) and UC2288 (1.0µM). Cells from spheroids were incubated with FGF2 plus inhibitors for 72 h before ALDEFLUOR/CD44 staining or counting and replating for 1st round of mammosphere culture for 7 days. The DMSO treatments are used as controls and set as fold one. **C-E**. TCGA ER + breast cancer patients data analysis for interactions in the generalized linear model between FGF2 (X-axis):FGFR1 expression and their impact on ssGSEA pathways related to stemness (**C-D**) and JAK-STAT (**E**) (Y-axis, *n* = 601). The three linear fit curves and 95% C.I. (grey shaded area) are shown to indicate the interaction effects between FGF2 and FGFR1 expression, where FGFR1 expression levels were grouped by tertiles (high *n* = 206; low *n* = 199; med *n* = 196)

Previous studies have indicated that FGF2, the FGFR signaling pathway, and the JAK-STAT pathway can amplify stemness traits in breast CSCs [36–39]. Additionally, p21 is linked to CSCs and serves as a biomarker for such cells [51]. To test if the role of FGF2 could shift from promoting proliferation to inducing a stem-like state in FGFR1 amplified cells, we next investigated the effect of FGF2 on cancer stemness-like traits using FACS analysis for ALDH and CD44 (two known markers of breast cancer stemness) [52]. FGF2 was shown to raise ALDH levels in MDA-MB-134 and T47D cells (Fig. S14A), while CD44 levels were elevated in all four cell lines (Fig. S14B). Additionally, FGF2 led to an increase in CSL (cancer stemness-like) /Live (live CSL cells versus all live cells) in all four cell lines, with a greater increase in the FGFR1 amplified cells (463.5 ± 86.7% in CAMA1 and 305.2 ± 20.5% in MDA-MB-134) compared to non-amplified cells (169.4 ± 42.2% in MCF7 and 172.3 ± 34.1%in T47D) (Fig. 7A). Both PD166866 and TAS-120 prevented FGF2-induced increases in ALDH levels and CD44 levels, as well as reduced the abundance of CSL/Live in all four cell lines (Fig. S14A-B, Fig. 7A). This suggests that FGF2 and FGFR1 amplification promoted cancer stemness. To demonstrate the role of JAK-STAT and CBP/p300 in regulating p21 and contributing to FGF2-promoted cancer stemness-like traits, we also evaluated the effect of UC2288, AZD1480, and SGC-CBP30 on reducing these traits within the same FACS analysis. As shown in Fig. S14A-B and Fig. 7A, UC2288 and SGC-CBP30 reduced ALDH and CD44 levels, and the proportion of cancer stem-like cells in all four cell lines. This result confirmed that the p21 and CBP contribute to FGFR-regulated cancer stemness. AZD1480 reduced CD44 levels and cancer stem-like cell abundance in most cancer cell lines. Although FGF2 enhanced stemness in FGFR1 amplified cells, this result indicates that JAK-STAT pathway contributes to stemness-like traits in both FGFR1-amplified and non-amplified cells.

Next, two cycles of mammosphere culture were performed to select stem-like cells enhanced by FGF2 treatment with their self-renewal ability. The relative percent change of mammosphere total area in both first-round culture (Fig. 7B) and second-round culture (Fig. S14C) revealed a significant increase in mammosphere formation due to FGF2 induction in CAMA1 and MDA-MB-134 cell cultures, while this promotional effect was absent in MCF7 and T47D cell cultures. PD186866, TAS-120, AZD1480, and SGC-CBP30 significantly reduced mammosphere total area in both rounds, while UC2288 exhibited inhibitory effects during the first round of culture.

To further test the role of FGF2 in promoting stemness-like traits, we assessed the transcription levels of key stemness marker genes, including CD24 and CD44 (Fig. S14D, Table S1). Employing qPCR experiments to determine the CDKN1A vs. CCND1 ratio (Fig. 3D), we observed that FGF2 upregulated CD44 in CAMA1, MDA-MB-134, and T47D, while TAS-120 attenuated the FGF2-induced increase. Conversely, CD24 was significantly inhibited by FGF2 treatment in MDA-MB-134 and MCF7, with a trend observed in CAMA1 (*p* = 0.17). TAS-120 reversed this inhibition in CAMA1 and MDA-MB-134 cells (Fig. S14D). To evaluate the impact of other inhibitors on stemness marker genes, an additional qPCR experiment was conducted using 3D cell culture with all four cell lines (Fig. S14E). FGF2 led to an elevation of CD44 in CAMA1 and MDA-MB-134, PD186866, AZD1480, UC2288, and SGC-CBP30 all demonstrated inhibitory effects in CAMA1 and MDA-MB-134, aligning with the results observed in FACS analysis and mammosphere culture. CD24 exhibited a trend of reduction with FGF2 treatment and an increase with PD186866 across all four cell lines, although some comparisons did not reach statistical significance. AZD1480 only reversed CD24 in MDA-MB-134, not in CAMA1, while UC2288 showed a reversal trend in both CAMA1 (*p* = 0.059) and MDA-MB-134 (*p* = 0.096).The combined use of FACS analysis and qPCR for assessing stemness features, along with mammosphere culture assays, affirmed that FGF ligands could enhance stemness-like traits through FGFR1-p21 regulation via the JAK-STAT pathway, contributing to stemness-like characteristics in FGFR1-amplified ER + breast cancer cells.

To validate this discovery in the TCGA ER + breast cancer patients, we analyzed the association between FGF2 and FGFR1 expression and pathway signature scores related to cancer stemness and JAK-STAT pathways. We observed significant interaction between FGF2 and FGFR1 with two stemness related signatures (BOOUEST_STEM_CELL UP *p* = 8.62 × 10^− 7^, LIM_MAMMARY_STEM_CELL_UP (*p* = 2.15 × 10^− 11^) (Fig. 7C-D), and the JAK/STAT signaling pathway (KEGG_JAK_STAT_SIGNALING_PATHWAY *p* = 4.15 × 10 − ^5^) (Fig. 7E). The positive interaction effects indicated the positive correlation between FGF2 with the stemness and JAK/STAT signatures were more pronounced in the high and medium FGFR1 groups compared to the low FGFR1 groups (Fig. 7C-E). These effects were also validated using the METABRIC dataset of ER + breast cancer patients. These results also showed a stronger correlation between FGF2 and the pathways related to cancer stemness (Fig. S15A-B) and JAK-STAT pathway (Fig. S15C) in FGFR1 high and medium groups compared to FGFR1 low groups, highlighting the importance of FGFR1 overexpression in enhancing cancer stemness and related pathways through FGF2.

## Discussion

The potential of FGFs and FGFRs as targets for cancer treatment has been recognized [4, 40, 53–55]. Despite the development and testing of various specific and pan-FGFR inhibitors in clinical trials, drug resistance remains a challenge [41]. Previous studies on aberrant FGFR in breast cancer focus on its promotion on resistance to endocrine therapy, CDK inhibitors and chemotherapy [10, 41, 56, 57]. However, the diverse effects of FGF and FGFR inhibitors in FGFR1 amplified and non-amplified tumors have not been well characterized, despite its important relevance to clinical use of these treatments.

**Fig. 8:**
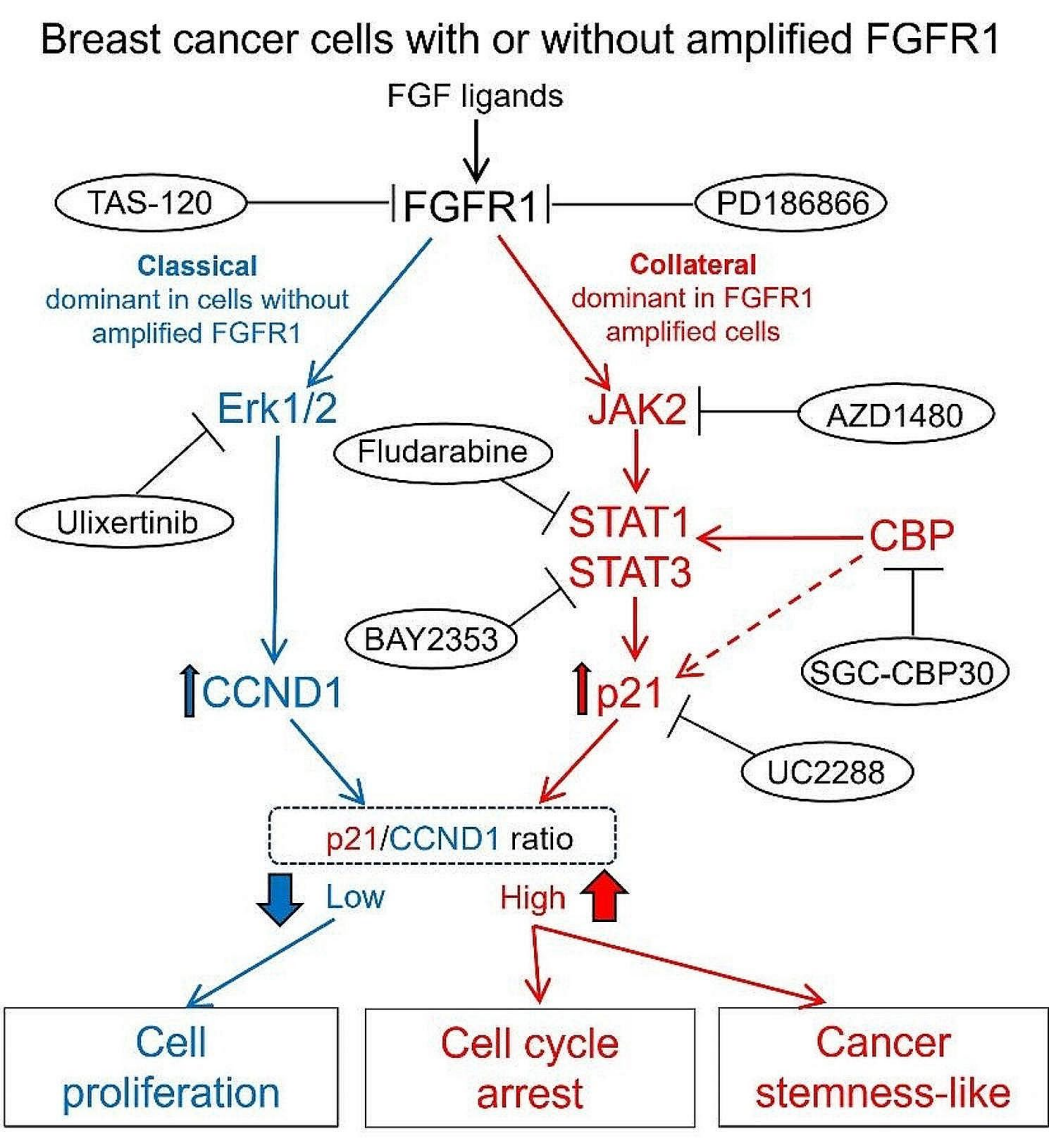
Brief figure summarizes the FGF2-FGFR1-p21 regulatory pathway in FGFR1 amplified ER + BC cells. FGF ligands can activate both the classical FGFR MAPK cascade to promote proliferation and a collateral JAK-STAT signaling to upregulate p21, which leads to a growth inhibitory effect and a stem cell-like state. In cancer cells with FGFR1 amplification, FGF ligands addition leads to enhanced upregulation of p21 and stem cell-like state. By using a range of inhibitors target different nodes of FGFR signaling, including FGFR1 (PD186866), pan-FGFR (TAS-120), JAK2 (AZD1480), STAT1 (Fludarabine), STAT3 (BAY2353), CBP/P300 (SGC-CBP30), ERK1/2 (Ulixertinib), and p21 (UC2288), these different states could be reversed

In this study, we compared the impact of FGF2 on cell growth and cancer stemness states in two groups of cancer cells, with and without FGFR1 amplification. We used a series of inhibitors to reverse the FGF2 effect on tumor growth, which elucidated paradoxical growth of cancer cells following FGFR1 inhibition in cells with FGFR1 amplification. We showed that p21 plays a key role in FGF2 induced reduction in cell proliferation with amplified FGFR1 by using p21 inhibitor UC2288, then showed specific FGFR1 inhibitor PD166866 could reverse the negative effects of FGF2, indicating an FGF2-FGFR1-p21 regulation pathways. Results with JAK2 and CBP/p300 inhibitors indicated that these pathways contribute to p21 regulation and cancer stemness trait induction. The results of our study indicate that amplified FGFR1 regulates a collateral JAK2/STAT signaling pathway that leads to increased levels of p21 and stemness traits, and that FGFR1 inhibition in these cells leads to a paradoxical increase in cell proliferation (Fig. 8). Important next steps include testing additional cells lines and patient samples to more broadly generalize effects of FGF ligands and FGFR inhibitors, and the importance of specific pathway nodes in driving proliferation and stemness.

The activation of canonical FGFR downstream MAPK signaling pathways promote tumor growth through CCND1 upregulation [21, 22]. As shown in Fig. 8, when FGFR1 is amplified, JAK-STAT with CBP/p300 as a potential coactivator is activated, which inhibits cancer progression through p21 upregulation. We examined the ratio of p21/CCND1 to evaluate proliferation promotion and inhibition signaling balances. In non-amplified cells (MCF7 and T47D), FGF2 induces low levels of activation of JAK-STAT pathway, and reduces the ratio of p21/CCND1, which resulted in cell cycle progression. In breast cancer cells with FGFR1 amplification (CAMA1 and MDA-MB-134), FGF2 elevated non-canonical FGFR downstream signaling pathways, especially JAK-STAT pathway, to promote p21 expression and modulate the ratio of p21/CCND1 to drive cell cycle arrest.

The data of ER + breast cancer patients from TCGA and METABRIC databases were analyzed using a generalized linear model in our study. The aim was to examine the relationship between FGF2 and FGFR1 and their impact on mRNA expression of P21 and CCND1, cell cycle status, and cancer stemness characteristics. Our results indicated that high FGFR1 groups had elevated p21/CCND1 ratios, reduced M and S phases, and heightened cancer stemness traits, while low FGFR1 groups demonstrated less impact or a contrary trend. Some of our experimental findings with cell line models, such as p21 levels, did not align with these analyses, potentially due to post-translational modifications and protein localization (such as p21 degradation and nuclear localization). Ultimately, our results support the hypothesis that FGFR1 level regulates the cell cycle and stemness status in response to FGF2 stimulation in breast cancer cells. FGFR1 levels can alter the state, and its combination with FGF2 determines FGF2’s ambivalent role in cell proliferation and stemness induction. Identifying FGFR1 levels in breast cancer samples could assist in predicting cell progression and choosing anti-FGFR inhibitors for future cancer treatment.

Our study demonstrated the ability of FGF ligands to stimulate cell proliferation and spheroid growth in ER + breast cancer cell lines without FGFR1 amplification (MCF7, T47D), a phenomenon widely recognized [21, 22]. Conversely, we also revealed a previously overlooked effect in cell lines with FGFR1 amplification (CAMA1, MDA-MB-134), where FGF ligands inhibited proliferation. Thus, our study has confirmed paradoxical or counterintuitive growth effects induced by FGF ligands, depending on FGFR1 amplification status.

Another noteworthy finding in our study is the inverse growth stimulatory and inhibitory effects FGFR inhibitors have in FGFR1 amplified and non-amplified ER + breast cancer cells. As anticipated, inhibitory effects were observed in cells without FGFR1 amplification, but surprisingly, promotional effects were observed in cells with FGFR1 amplification. This unexpected outcome, particularly in microenvironments with elevated FGF ligand levels [58], could have detrimental clinical effect in patients, such as ER + breast cancer patients with high FGFR1 and FGF2 expression. The paradoxical growth effects may help explain the failure of clinical trials with FGFR inhibitor as monotherapy in breast cancer patients with FGFR1 amplification [42, 58, 59]. Future efforts that consider combination therapeutic strategies may overcome the limitations of single-agent FGFR inhibition in FGFR1 amplified cancers, might serve as a potential clinical approach for enhancing efficacy and minimizing unexpected outcomes [58].

In contrast to earlier studies concentrating solely on either inhibitory or promotional growth impacts of FGF ligands and their mechanisms on specific cell lines [18, 20–22], our research took a different approach aiming to unravel the reasons behind the dual promotional and inhibitory effects of FGF ligands in ER + breast cancer. By comparing the growth effects of FGF ligands on two subsets of ER + breast cancer cell lines: one with FGFR1 amplification (CAMA1, MDA-MB-134) and the other without (MCF7, T47D), our study not only validated the paradoxical growth effects across various FGF ligand types, but also delved into the mechanisms underlying these paradoxical growth effects. We unveiled the dynamic role of FGF ligands, shifting from promoting proliferation to inducing a stem-like state through the regulation of the CDKN1A/CCND1 ratio via the JAK-STAT pathway in the presence of amplified FGFR1. In the meanwhile, our research provided correlation analyses between FGF2-FGFR1 and downstream pathways, utilizing patient databases, and underscored the potential adverse consequences associated with the monotherapy of FGFR inhibitors in clinical treatments for ER + breast cancer.

Three-dimensional spheroid culture model was used in this study instead of two-dimensional (2D) culture model because the use of 3D spheroid culture provides several advantages compared to conventional 2D cell cultures: (i) The 3D structure of spheroids and organoids more closely mirrors the intricate nature of tumors, with a mixture of cells in different stages of proliferation and a necrotic core with fluctuating distributions of oxygen and nutrients. This complexity makes drug testing with 3D spheroid cultures more relevant to tumor growth [60, 61]; (ii) In contrast to 2D culture, where only surviving cells are counted after drug treatment, leading to limited calculations of cell proliferation, 3D culture maintains all cells throughout the drug treatment period, allowing for more comprehensive cell count data through measurement of spheroid size; and (iii) Culturing spheroids in a 3D environment provides various benefits for detecting cancer stem cells (CSCs). This effect is due to the variations in hypoxic and growth regions in the spheroid [36].

Although the FGFR1/MEK/ERK pathway has been shown to promote stemness in both Triple-negative breast cancer (TNBC) cell line MDA-MB-231 [62] and luminal A breast cancer cells [63], there are other mechanisms that can be considered. JAK-STAT signaling plays a crucial role in cancer stemness by increasing stemness and enhancing tumor progression through the induction of epithelial–mesenchymal transition [64]. Furthermore, inhibition of JAK-STAT1/3 signaling has been found to suppress CSC capabilities both in vitro and in vivo [65]. The crosstalk between the JAK/STAT pathway and other pathways, such as the formation of complexes between STAT3/SMAD3 and STAT3/CD44, contributes to the generation of cancer stem cells [66]. The level of p21 expression has also been linked to cell quiescence or mature cell development, and p21 itself could serve as a biomarker for cancer stem cells [51]. In our study, we used various inhibitors to show that the JAK-STAT pathway regulates p21, contributing to the promotion of cancer stemness traits through FGF2-FGFR1, in addition to their regulation of cell proliferation.

## Conclusions

In summary, our study has demonstrated that FGFR1 amplification in ER + breast cancer cells activate a collateral JAK-STAT pathway and leads to p21 upregulation, which inhibits the proliferation of cancer cells and enhances their stemness properties with FGF2 simulation. This collateral signaling pathway then paradoxically leads to increased cancer cell growth in FGFR1 amplified cells treated with FGFR1 inhibitors and highlights a challenge of FGFR inhibitor use in cancer treatment.

### Electronic supplementary material

Below is the link to the electronic supplementary material.

Supplementary Material 1

## Data Availability

All genomic and transcriptomic data used in this study are publicly available. Gene expression, copy number, somatic variants and annotations data for TCGA breast cancer patients are available through the Xena platform (https://xenabrowser.net/) under the TCGA BRCA hub. Gene expression and annotations data for METABRIC patients are available through the cBioPortal (http://www.cbioportal.org/study/summary?id=brca_metabric). Custom scripts for the generalized linear models analysis are available via GitHub (https://github.com/U54Bioinformatics/FGF2_FGFR1_2023).

## Abbreviations

BC: Breast cancer
ER: Estrogen receptor
TNBC: Triple-negative breast cancer
FGF: Fibroblast growth factor
FGFR: Fibroblast growth factor receptor
CDKs: Cyclin-dependent kinases
JAK: Janus kinase
2D: Two-dimensional
3D: Three-dimensional
STAT: Signal transducer and activator of transcription
MAPK: Mitogen-activated protein kinase
CBP: Cyclic AMP response element-binding protein (CREB)
PI3K: Phosphoinositide 3-kinase
CSL: Cancer stemness-like
TCGA: The Cancer Genome Atlas
METABRIC: Molecular Taxonomy of Breast Cancer International Consortium
