# Supplementary material for: Paradoxical cancer cell proliferation after FGFR inhibition through decreased p21 signaling in FGFR1-amplified breast cancer cells
Source: Breast Cancer Res. 2024 Mar 29;26:54. doi: 10.1186/s13058-024-01808-7 (PMC10979625; doi:10.1186/s13058-024-01808-7)

# Supplementary Methods

Assessing FGF ligand effects using cancer growth experiments

The results of all experiments were normalized to the DMSO control treatment for each cell line. To conduct 3D spheroid imaging experiments, cells were plated in black walled, 96-well round-bottom ultra-low attachment spheroid microplates (Corning Cat. No.: 4520) at a density of 5,000 cells per well in a volume of 200 µL of medium. After 18-24 hours, spheroids were imaged, a 100 µL of medium containing 2x the concentration of the drug being tested was replaced. The spheroids were treated with the drugs as indicated for up to 14 days, with imaging and medium/drug changes occurring every 3 or 4 days. Spheroids were then treated every three or four days and concurrently imaged on 5 occasions (Days: 1, 4, 7, 10,14).

Quantifying cell abundance during time course experiments

Cancer cell population growth was quantified under differing treatment conditions, by measuring spheroid area and its brightfield intensity during the growth of replicates across each experiment time point (as described previously by Grolmusz et al [1]. In brief, imaging was performed using Cytation 5 imager (Biotek Instruments) to gather brightfield signal intensity. Raw data processing and image analysis were performed using Gen5 3.05 and 3.10 software (Biotek Instruments). Briefly, the stitching of 2 × 2 montage images and Z-projection of six layers using focus stacking was performed on raw images followed by threshold-based spheroid area analysis.

Spheroid area ($A$) and the mean brightfield pixel intensity within the spheroid area ($\bar{I}$) were jointly used to quantify each cell type’s abundance over time after constructing an experimental standard curve to map known cell abundances of each cell type to measurements of these two quantities 24 h after plating (see images below). The experimentally observed relationship between cell number and the spheroid’s area and brightfield intensity was characterized (R^2^=0.98 across cell lines) by a generalized additive model of the form:

$$E\left( log10(Cell Number) \right)=B_{0}+B_{A}*log10(A)+ f_{I}\left( log10(\bar{I} \right))$$

Here, $B_{0}$ captures the limit of detectability of cancer cells, $B_{1}$ is the power function exponent relating spheroid area to cell number and $f_{I}$ is an estimated smooth non-linear function capturing the negative relationship between the spheroid’s thickness and the mean brightfield light intensity transiting through it. Cross validation (k fold, leaving one replicate out) confirmed that spheroid area and brightfield intensity accurately predicted cell numbers in out of sample populations (CV R2: mean=0.97, sd=0.004, n=3)."The parametrized model was then used to infer cell counts for each replicate cancer population during treatment based on spheroid area and its mean brightfield intensity measured from experimental imaging.


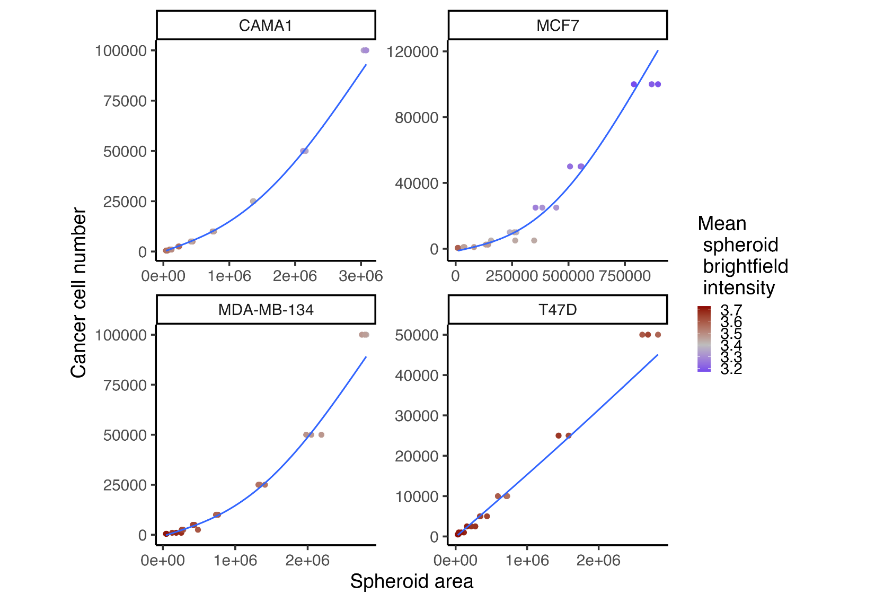

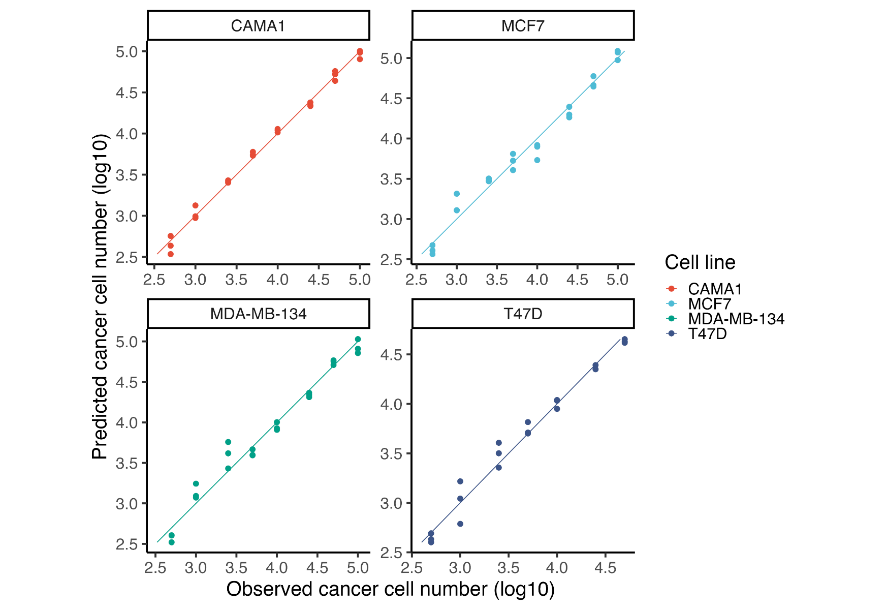


Characterizing cancer growth trajectories across treatments

For each replicate cancer population, we quantified its speed of growth or shrinkage over the 14-day period. Specifically, the relative growth rate (rgr; described in [2] of each cancer population was used to account for initial variation in spheroid abundance and determined the average hourly change in log cancer abundance between the start ($t_{0}$) and end of treatment ($t_{max}=day 14$), calculated as:

$rgr=\frac{\log\left( N\left( t_{max} \right) \right)-log(N\left( t_{0} \right))}{t_{max}-t_{0}}$ .

We analyzed how the cancer growth trajectory (log($N\left( t \right))$) of each of the four cell lines was impacted by the individual and combined effects of and FGF ligand addition and targeted inhibitor treatment, using generalized additive models (GAMs). For each of the four cell lines, the fitted GAM characterized treatment impacts over time, with 95% confidence intervals showing timepoints when cancer population diverged during treatment. Models were fitted and significance determined using the mgcv r package (version 1.9-0). No significant difference in cell counts were detected between treatment groups prior to treatment (log($N\left( t_{0} \right)\mathrm{ANOVA}$). The impacts of FGF ligand addition and targeted inhibitors on each cell line’s abundance was determined by calculating the log fold change in post-treatment cell counts (log($N\left( t_{max} \right))$) of treated replicates compared with those of DMSO controls. We then identified significant treatment effects, using ANOVA to partition variability of cell counts across replicate. The student’s T-test was employed to assess the comparison between the treatment with and without FGF2 within the identical inhibitor dose group.

Immunoblotting

Proteins were extracted from cells using the RIPA Lysis Buffer (Thermo Scientific) that contained 1mM PMSF and a Halt^TM^ protease and phosphatase inhibitor cocktail (Thermo Scientific). The protein concentration in the extracted lysates was determined using the BCA assay (Pierce). Equal amounts of denatured proteins were then separated using SDS-PAGE (4% to 20% Tris-Glycine Gel from Bio-Rad) and visualized using a chemiluminescence procedure (Thermo Scientific) with a standard protocol. The separated proteins were then immunoblotted overnight using specific antibodies against target proteins such as p21 Waf1/Cip1 (12D1) (#2947), CCND1 (92G2) (2978), pSTAT1 (Tyr701) (58D6) (#9167), STAT1 (9H2) (#9176), pSTAT3 (Tyr705) (D3A7) (#9145), STAT3 (79D7) (#4904), JAK2 (#3230), CBP (D986)(#7425), p-p44/42 MAPK (Erk1/2) (Thr202/Tyr204) (#4370) from Cell Signaling, β-actin (C4) (sc-47778) from Santa Cruz Biotechnology, FGFR1 Antibody (EPR806Y) (ab76464) from Abcam, pJAK2 (Tyr1007/1008) ([07-606](https://www.emdmillipore.com/US/en/product/Anti-phospho-JAK2-Tyr1007-1008-Antibody,MM_NF-07-606)) from Millipore Sigma.

Quantitative real time-PCR analysis

RNA extraction was performed using the RNeasy Mini kit from QIAGEN. cDNA synthesis was then carried out utilizing the Maxima First Strand cDNA Synthesis Kit from Thermo Scientific. The resulting cDNA was analyzed via quantitative real-time PCR (qRT-PCR) using the Fast SYBR Green Master Mix (Applied Biosystems, Thermo Scientific) as per the manufacturer's protocol. The mRNA expression levels of various genes were determined relative to RPLP0, with primer sequences detailed in Table S1.

**Table S1. The primer sequences used in RT-qPCR assay.**

| Genes | Forward Primer | Reverse Primer |
| --- | --- | --- |
| RPLP0 | AGCCCAGAACACTGGTCTC | ACTCAGGATTTCAATGGTGCC |
| FGFR1 | CCCGTAGCTCCATATTGGACA | TTTGCCATTTTTCAACCAGCG |
| CDKN1A | TGTCCGTCAGAACCCATGC | AAAGTCGAAGTTCCATCGCTC |
| CCND1 | GCTGCGAAGTGGAAACCATC | CCTCCTTCTGCACACATTTGAA |
| CD24 | CTCCTACCCACGCAGATTTATTC | AGAGTGAGACCACGAAGAGAC |
| CD44 | CTGCCGCTTTGCAGGTGTA | CATTGTGGGCAAGGTGCTATT |

Co-transduction/transfection

Two vectors were purchased from Generscript: the empty pCMV6-XL4 vector (used as a control) and the pCMV6-XL4-FGFR1 vector. Vector DNA was amplified within DH5α and extracted using EndoFree Plasmid Maxi Kit (Qiagen No. 12362). Overexpression of FGFR1 in breast cancer cells was achieved in 2D culture within 6-well plate (Corning No. 3516) using Lipofectamine3000 (Thermo Scientific) according to the manufacturer's instructions. Transfected cells were harvested 48 hours post-transfection using trypsin, counted, and subsequently utilized for immunoblot analysis and 3D spheroid culture. Equal numbers of FGFR1 and control vector transfected live cells (5000 cells per well) were seeded in 96-well round-bottom ultra-low attachment spheroid microplates (Corning No. 4520).

Cell cycle analysis

Flow cytometric analysis of cell cycle was conducted using propidium iodide DNA staining, following the protocol from Abcam. The cells were first washed and suspended in cold PBS, then fixed in ice-cold 70% ethanol for overnight incubation at 4°C. Afterwards, the cells were treated with RNase A for 30 minutes at room temperature, stained with propidium iodide at a concentration of 50 µg/mL, and subjected to FACS analysis at 605 nm for propidium iodide. The cell cycle analysis was performed in triplicate on all samples using Flowjo V10.3 to determine the percentage of cells in each stage of the cell cycle.

Flow Cytometry for cancer stemness

The organoids were collected using centrifugation (300g, 5 min) and dissociated using trypsin-EDTA (5 min, 37°C). The cells were then stained with ALDEFLUOR (1:100 dilution, STEMCELL TECHNOLOGIES, 30 min, 37°C) and anti-CD44-PE antibody (1:50 dilution, Miltenyi, 25 min, on ice). Negative controls for ALDEFLUOR were obtained by using the DEAB inhibitor without CD44-PE staining. Each sample was conducted using 100 µL of ALDEFLUOR Assay as the staining solution, followed by CD44 staining within the ALDEFLUOR Assay Buffer (100 µL). DAPI (final concentration of 1 µg/mL) was added to label dead cells before FACS analysis. All procedures were done in triplicate. FACS gating and analysis were described in Chi et al [3]. The CSL population (ALDH+ and CD44+) was evaluated as a percentage of CSL vs live cells (DAPI negative).

Mammosphere formation assay

Mammosphere assay was performed following the protocol mentioned previously with modification [3-4]. Cells were plated in 96-well spheroid plates with black walls (Corning 4520) at 40,000 per/well in 1200µL medium. After 18-24 hours, 100µL medium with 2x FGF2 protein or drug was replaced at final concentration for FGF2 25ng/mL or FGF2 plus PD186866 1.0 µM, TAS-120 1.0 µM, AZD1480 0.2 µM, SGC-CBP30 10 µM, and UC2288 1.0 µM), respectively. After 72 hours, the spheroids were harvested from 12 wells, and dissociated with Trypsin for 5min at 37°C. The cells were pelleted, counted, and resuspended in 200µL mammosphere culture media (500mL phenol-free RPMI plus 20mL B27, plus human epidermal growth factor 20ng/mL, 1% antibiotic–antimycotic solution, all from Fisher Scientific). For each treatment, the cells were seeded at 2500 cells per well in 24 well plates (ultralow attachment from Corning No. 3473) with 1.0 mL mammosphere culture media supplied with DMSO only (for cells with DMSO and FGF2 treatment) or inhibitors mentioned above without FGF2 (for cells with FGF2 plus inhibitor treatment) in triplicates. After 7 days in culture, the mammosphere colonies were imaged with a Zeiss Axio Observer 7 microscope from triplicate wells. For the second mammosphere culture, the mammosphere colonies with the same treatment were collected and incubated with Trypsin for 5min at 37°C, followed by 10 passages through a 22-gauge needle for disassociation. The live cells were counted and seeded again at 2500 per well in 24 well ultralow attachment plate with 1.0 mL media supplied with DMSO or inhibitors in triplicates as the same conditions used in the first mammosphere culture. After another 7 days in culture, the mammosphere colonies were imaged again with a Zeiss Axio Observer 7 microscope from triplicate wells. All the mammosphere colonies (with diameters of ≥60 µm) were selected and counted using Qupath software (version 0.4.4) for total area measurement.

TCGA and METABRIC patients’ data analysis

Gene expression data and annotations from TCGA breast cancer patients were retrieved using the Xena platform [5], [Gene expression data and annotations from METABRIC] 6] were obtained using the cBioPortal (<http://www.cbioportal.org/study/summary?id=brca_metabric>).  TCGA RNAseq data was collected as RSEM normalized counts, transformed using log2(x+1), and somatic mutations, including SNPs and indels, were retrieved as MC3 calls. METABRIC microarray data generated using the Illumina HT-12 v3 platform were retrieved after beadarray normalization and log2 transformation. We used generalized linear models to analyze the associations between *FGF2* and *FGFR1* expression, including interaction effects, with levels of *CCND1*, *CDKN1A*, and the ratio of *CDKN1A* to *CCND1*. The impact of TP53 mutations on these associations with *FGFR1* were also evaluated by including mutation status as a covariate in this model.

To evaluate the associations and interaction effects between *FGF2* and *FGFR1* expression and cell cycle states, we used the single sample gene set enrichment (ssGSEA) scores of the REACTOME signatures for various cell cycle phases calculated using the GSVA package for R [7]. Similarly, we used the ssGSEA scores of various stemness signatures from the Molecular Signatures Database (MSigDb) [8] curated (C2) gene set to investigate the associations with *FGF2* and *FGFR1* expression. For both the cell cycle and stemness signature analyses, we used generalized linear models with an interaction term for *FGF2* and *FGFR1*. To illustrate the directionality of the interaction effect, we stratified the samples from TCGA and METABRIC cohort based on *FGFR1* expression tertiles and reported the P-values of the interaction term. Results with a p-value of less than 0.05 were considered statistically significant. All analyses were conducted using R version 4.05.

# Supplementary References

1. Grolmusz VK, Chen J, Emond R, Cosgrove PA, Pflieger L, Nath A, et al. [Exploiting collateral sensitivity controls growth of mixed culture of sensitive and resistant cells and decreases selection for resistant cells in a cell line model.](https://pubmed.ncbi.nlm.nih.gov/32565737/) Cancer Cell Int. 2020; 20:253.
2. Hoffmann WA, Poorter H. Avoiding bias in calculations of relative growth rate. Annals of botany. 2002; 90:37-42. PMID: 12125771
3. Chi F, Liu J, Brady SW, Cosgrove PA, Nath A, McQuerry JA, et al. [A `one-two punch' therapy strategy to target chemoresistance in estrogen receptor positive breast cancer.](https://pubmed.ncbi.nlm.nih.gov/33221681/) Transl Oncol. 2021; 14:100946.
4. Montales MT, Rahal OM, Kang J, Rogers TJ, Prior RL, Wu X, et al. [Repression of mammosphere formation of human breast cancer cells by soy isoflavone genistein and blueberry polyphenolic acids suggests diet-mediated targeting of cancer stem-like/progenitor cells.](https://pubmed.ncbi.nlm.nih.gov/22219179/) Carcinogenesis. 2012;33:652-60.
5. Goldman MJ, Craft B, Hastie M, Repečka K, McDade F, Kamath A, et al. [Visualizing and interpreting cancer genomics data via the Xena platform.](https://pubmed.ncbi.nlm.nih.gov/32444850/) Nat Biotechnol. 2020; 38:675-678.
6. Ellrott K, Bailey MH, Saksena G, Covington KR, Kandoth C, Stewart C, et al. [Scalable Open Science Approach for Mutation Calling of Tumor Exomes Using Multiple Genomic Pipelines.](https://pubmed.ncbi.nlm.nih.gov/29596782/) Cell Syst. 2018; 6:271-281.
7. Hänzelmann S, Castelo R, Guinney J.  [GSVA: gene set variation analysis for microarray and RNA-seq data.](https://pubmed.ncbi.nlm.nih.gov/23323831/) BMC Bioinformatics. 2013; 14:7.
8. Liberzon A, Subramanian A, Pinchback R, Thorvaldsdóttir H, Tamayo P, Mesirov JP. [Molecular signatures database (MSigDB) 3.0.](https://pubmed.ncbi.nlm.nih.gov/21546393/) Bioinformatics. 2011; 27:1739-40.

# Supplementary Figures


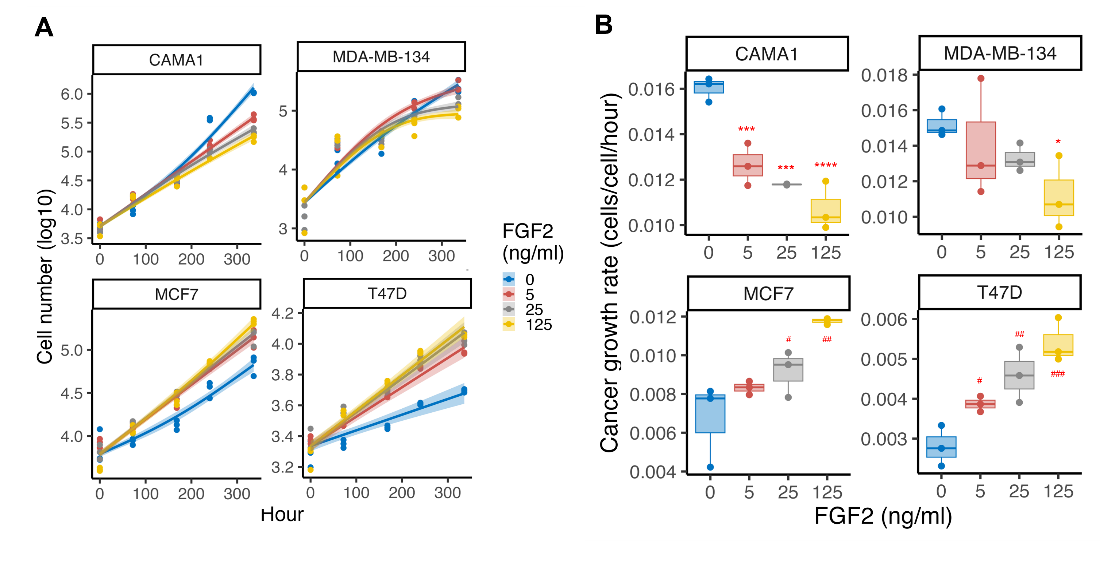


**Figure S1. FGF2 induced paradoxical growth effects in FGFR1 amplified and non-amplified ER+ cells**. **A**. Time course of cell number change over 14 days 3D culture for FGFR1 amplified cell lines (CAMA1 and MDA-MB-134) and non-amplified cell lines (MCF7 and T47D) show paradoxical growth effects of FGF2 simulation with various doses (0, 5, 25, 125 ng/mL). B. Cancer cell growth rate within spheroids under increasing doses of FGF2. An '*' denotes cells with treatment significantly lower than those without treatment (p<0.05), while a '#' indicates cells with treatment significantly higher than those without treatment (p<0.05).


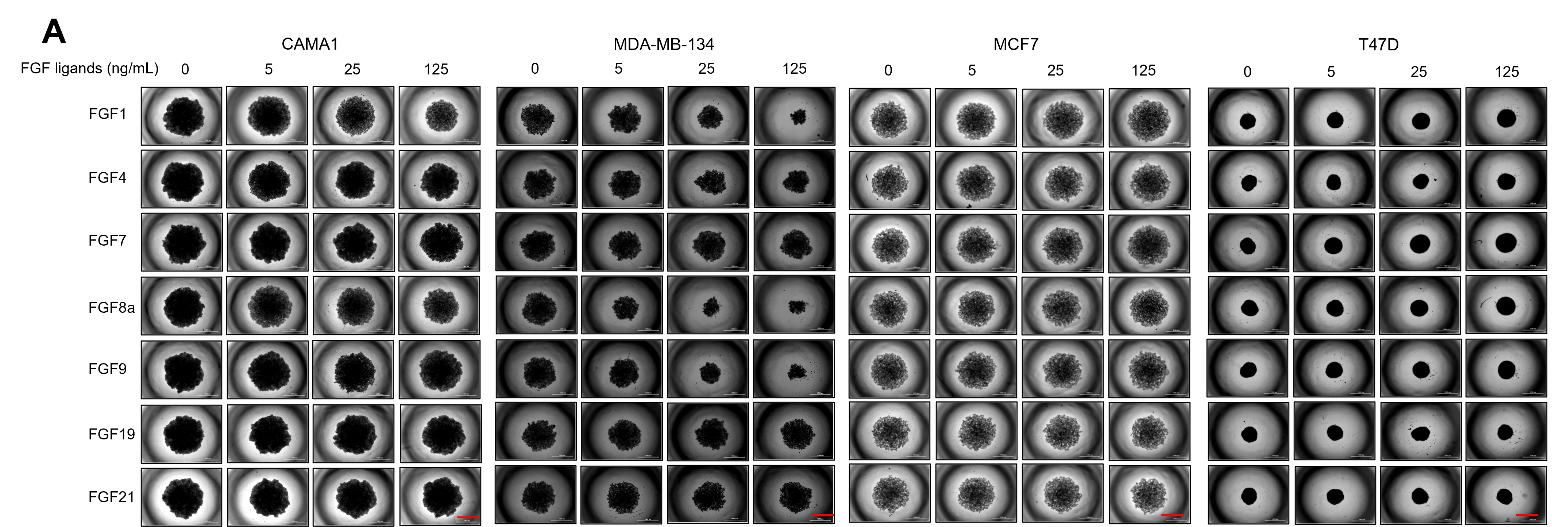


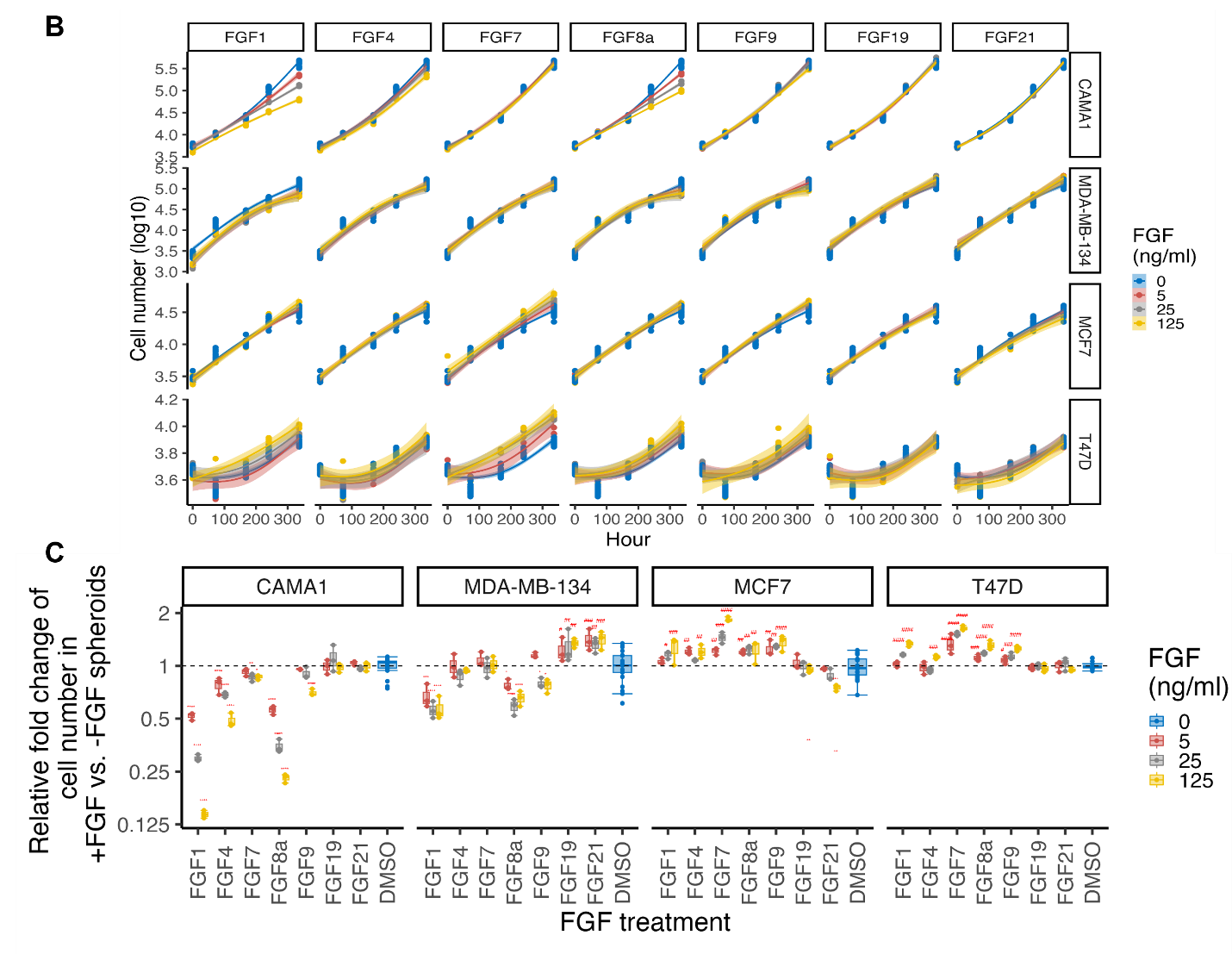


**Figure S2. Other FGF ligands induced paradoxical growth effects in FGFR1 amplified and non-amplified ER+ cells**. **A-C.** Spheroid images of FGFR1 amplified cells (CAMA1 and MDA-MB-134) and non-amplified cells (MCF7 and T47D) are shown after incubation with different FGF ligands (including FGF1, FGF4, FGF7, FGF8a, FGF9, FGF19 and FGF21) for 14 days at various doses (0, 5, 25, 125 ng/mL). Bar equals 1000 µm.  **B**. Time course of cell number change over 14 days 3D culture for two groups of cell lines in Panel **A.** **C**. Relative fold change of cell number in +FGF2 vs. -FGF2 spheroids in Panel **A** are depicted, with the DMSO control group serving as controls and set to a fold of one. An '*' denotes cells with treatment significantly lower than those without treatment (p<0.05), while a '#' indicates cells with treatment significantly higher than those without treatment (p<0.05).


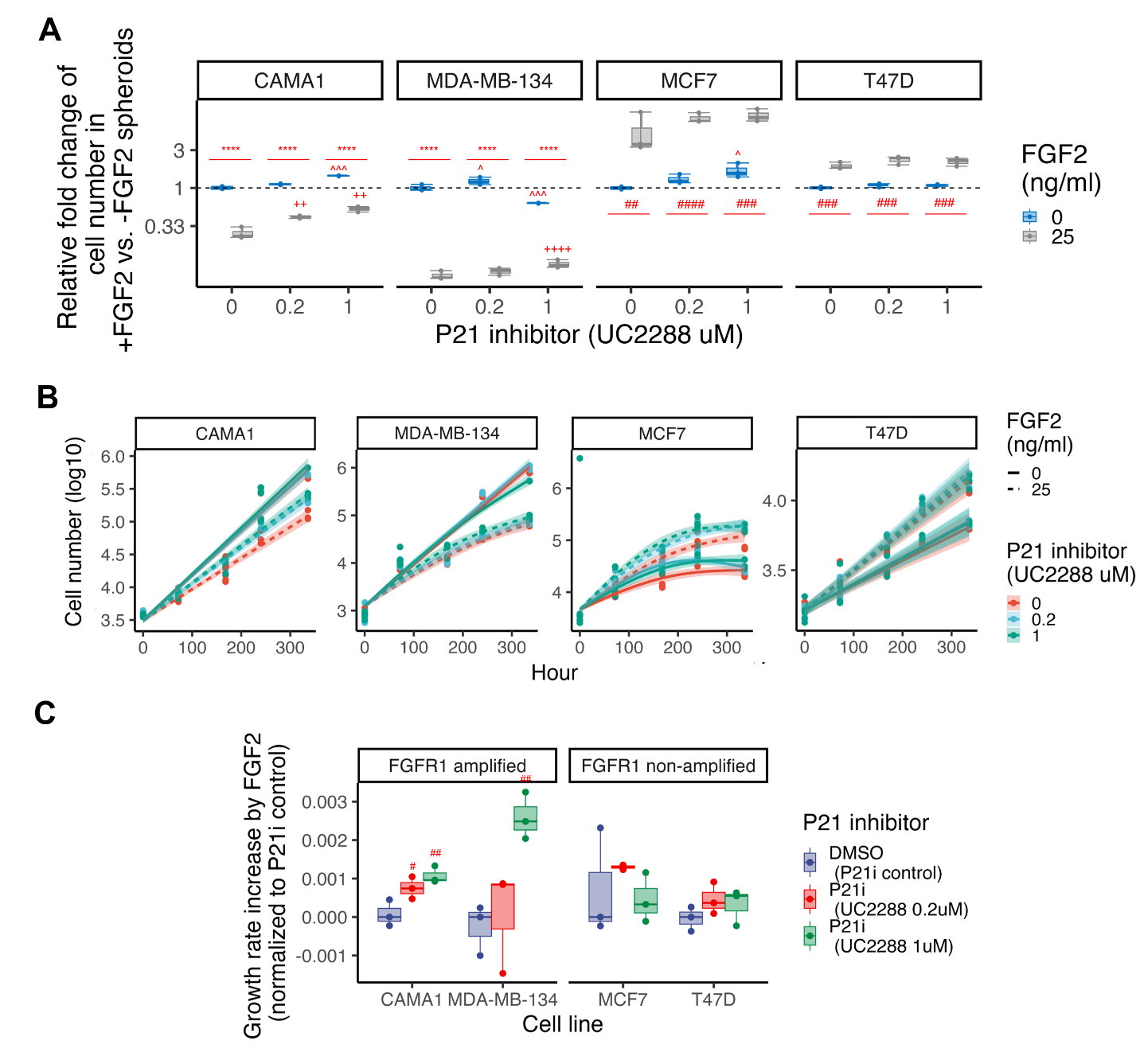
**Figure S3. p21 inhibitor UC2288 reversed FGF2 induced paradoxical proliferation. A**. Relative fold change of cell number in +FGF2 vs. -FGF2 spheroids for images in **Fig. 1F**, with DMSO only treatments serving as controls and set to a fold of one. **B**. Time course of cell number change over 14 days 3D culture for two groups of cell lines in **Fig. 1F. C**. Cancer cell growth rate normalized to inhibitor only in each dose group. DMSO treatments serve as the controls in each cell line.


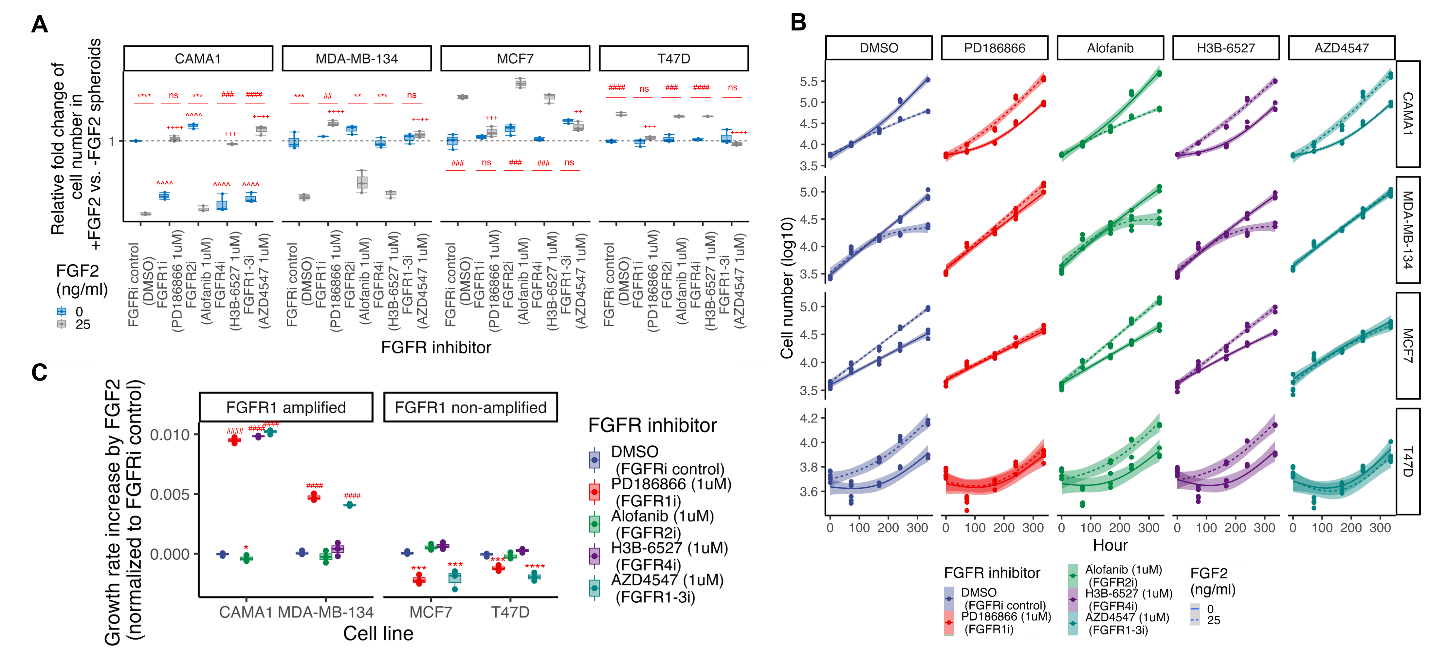
**Figure S4. The paradoxical proliferation caused by FGF2 can be reversed by specific FGFR inhibitors** **A**. Relative fold change of cell number in +FGF2 vs. -FGF2 spheroids for images in **Fig. 2A** are depicted, with the DMSO control group serving as controls and set to a fold of one. **B**. Time course of cell number change over 14 days 3D culture for two groups of cell lines in **Fig. 2A. C**. Cancer cell growth rate normalized to inhibitor only in each inhibitor group. DMSO treatments serve as the controls in each cell line.


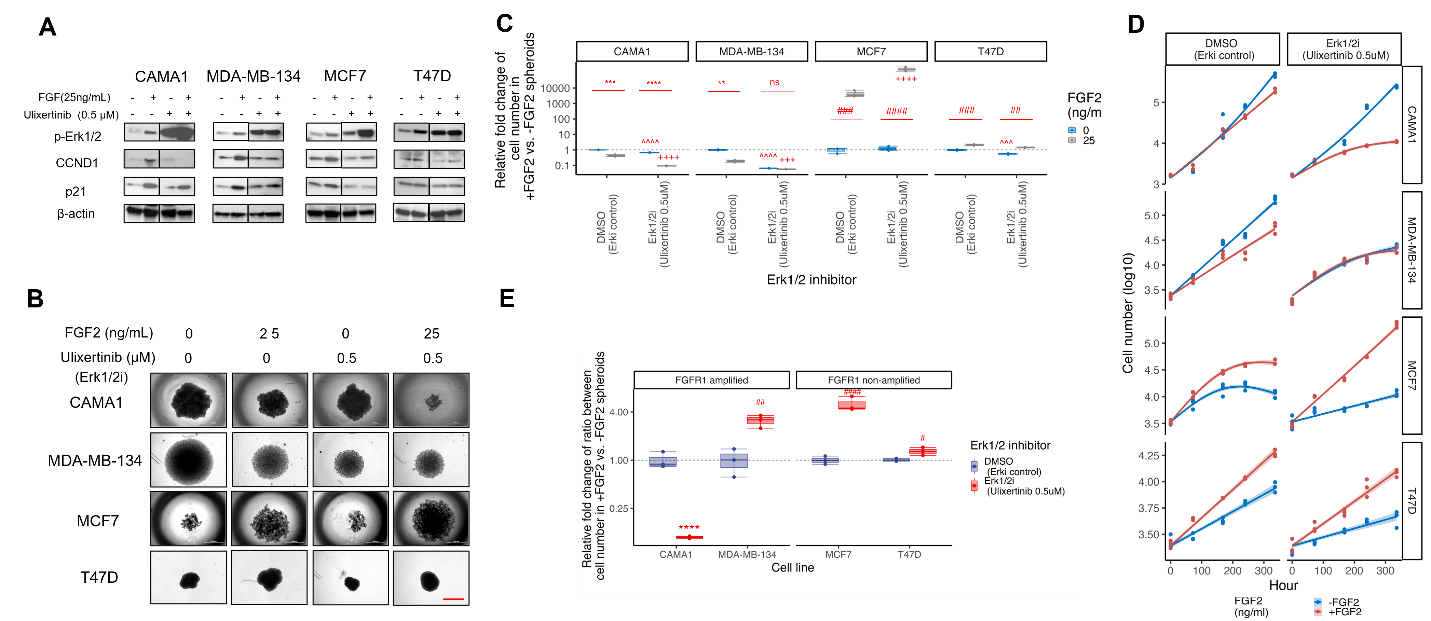
**Figure S5. Erk1/2 inhibitor Ulixertinib could not reverse the paradoxical proliferation caused by FGF2. A.** Immunoblotting shows the effect of Ulixertinib (0.5 µM) with FGF2 on CCND1 and p21protein levels and activation of Erk1/2 in 3D cell cultures after 72 hours of treatment with FGF2 (25 ng/mL). Bar equals 1000 µm. **B**. Spheroid images show the effects of irreversible Erk1/2 inhibitor Ulixertinib (0.5 µM) on FGFR1 amplified cells (CAMA1 and MDA-MB-134) and non-amplified cells (MCF7 and T47D) with and without FGF2 treatment (25 ng/mL) for 14 days. **C**. Relative fold change of cell number in +FGF2 vs. -FGF2 spheroids for images in panel **B** are depicted, with the DMSO control group serving as controls and set to a fold of one. **D**. Time course of cell number change over 14 days 3D culture for two groups of cell lines in panel **B**. **E**. Cancer cell growth rate normalized to inhibitor only in each inhibitor group. DMSO treatments serve as the controls in each cell line.


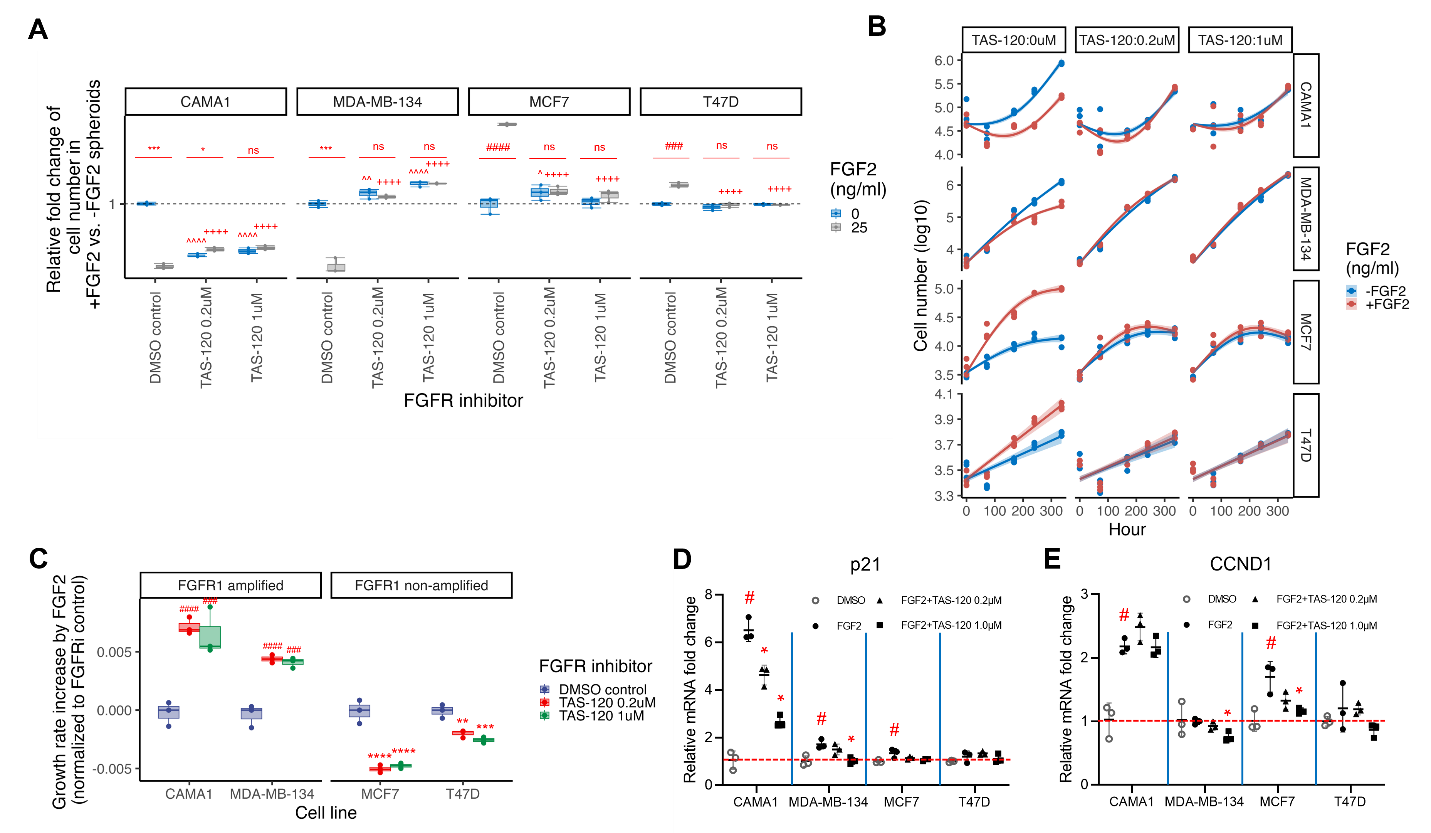
**Figure S6. TAS-120 inhibited FGF2 increased p21 level in both FGFR1 amplified ER+ BC cells. A**. Relative fold change of cell number in +FGF2 vs. -FGF2 spheroids for images in **Fig. 3A** are depicted, with the DMSO control group serving as controls and set to a fold of one. **B**. Time course of cell number change over 14 days 3D culture for two groups of cell lines in **Fig. 3A.** **C**. Cancer cell growth rate normalized to inhibitor only in each dose group. DMSO treatments serve as the controls in each cell line. **D-E.** The mRNA expression levels of p21 (A) and CCND1 (B) were measured in ER+ cell 3D cultures after incubation with FGF2 (25ng/mL) and TAS-120 at different doses (0.2 and 1.0 µM) for 72 hours. The mRNA expression levels are normalized to RPLP0 levels, and the control treatments with DMSO only are set as a fold of one.

**
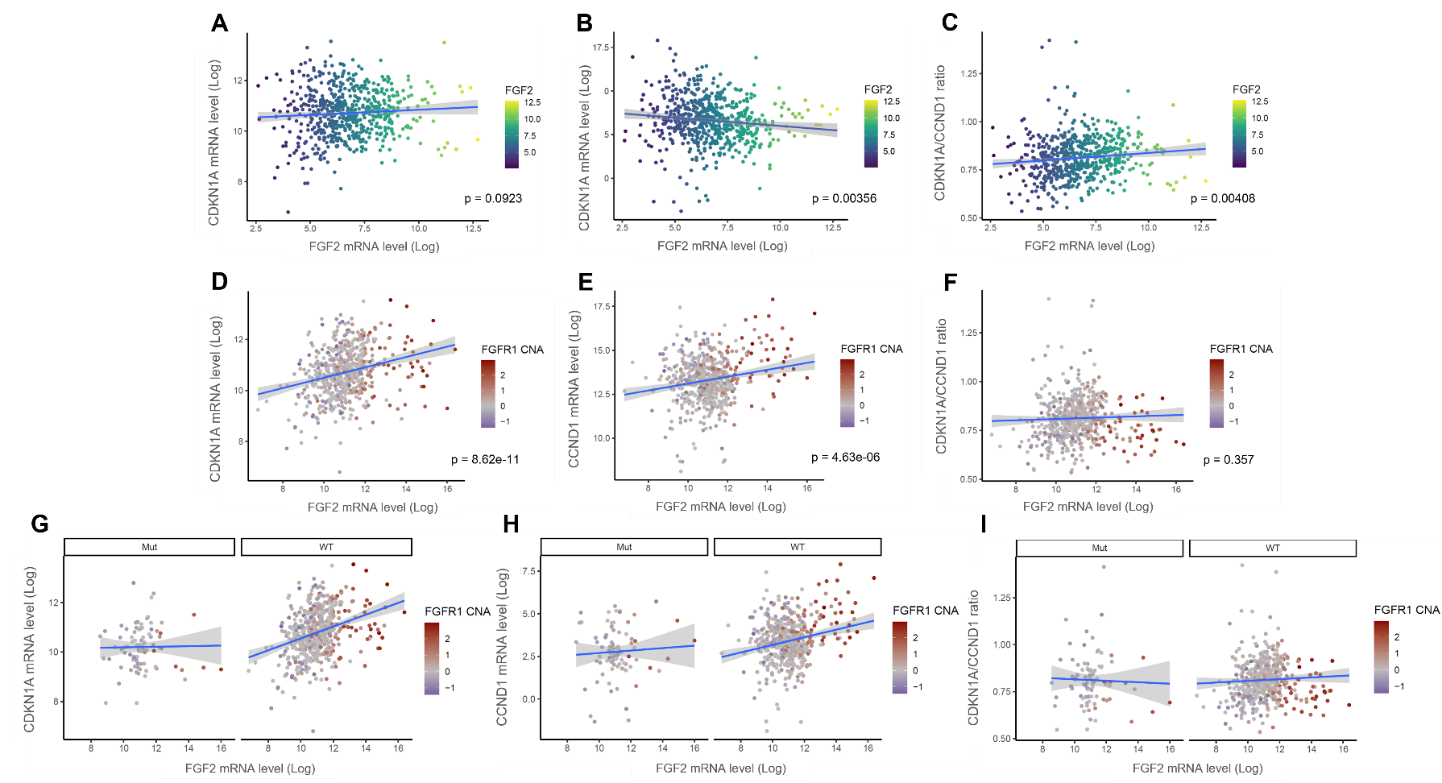
**

**Figure S7. Comparison of FGF2 with CDKN1A, CCND1, and CDKN1/CCND1 ratio in TCGA ER+ BC patients. A-C.** Plots displaying linear fit curves comparing FGF2 expression (X-axis) with CDKN1A (**A**), CCND1 (**B**), and CDKN1/CCND1 ratio (**C**) (Y-axes) in TCGA ER+ breast cancer patients (n = 601). **D-F**. Linear fit curves for FGF2 expression (X-axis) with CDKN1A (**D**), CCND1 (**E**), and CDKN1/CCND1 (**F**) ratio on the Y-axes. **G-I**. Linear fit curves in TCGA samples stratified by TP53 mutation status (WT n = 513; mut n = 88) showing the association between FGF2 expression (X-axis) with CDKN1A (**G**), CCND1 (**H**), and CDKN1A/CCND1 ratio (**I**) (Y-axes). Individual points are colored to reflect FGFR1 copy number status, as indicated in the legends in **D-I**.


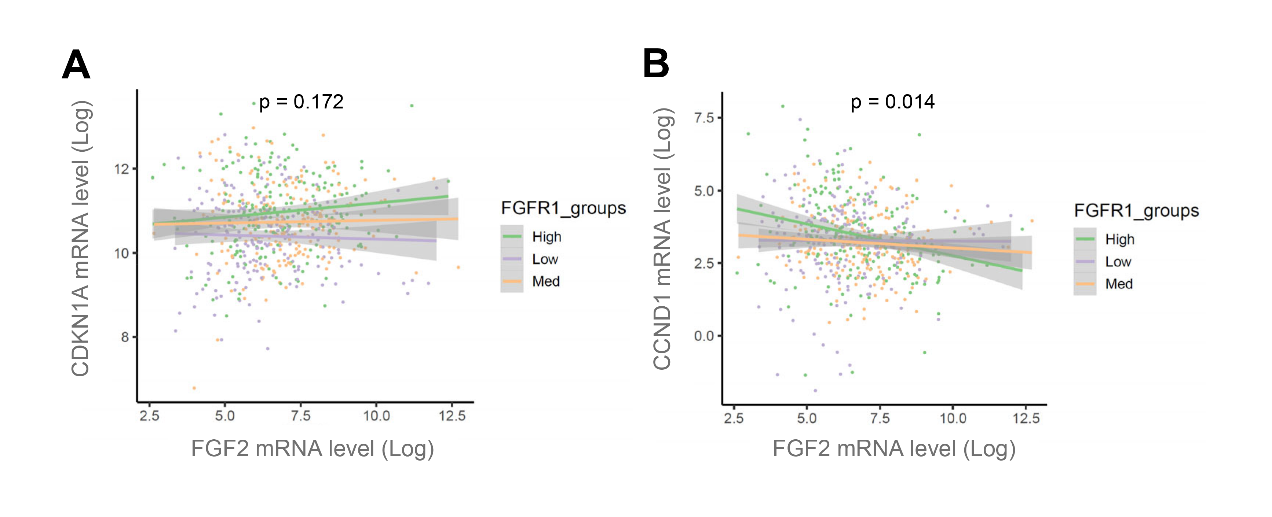


**Figure S8. Relationship analysis between FGF2 expression levels of and CDKN1A/CCND1 in ER+ BC patients from TCGA. A-B.** Plots that depict the relationship between the expression levels of FGF2 (X-axis) and the CDKN1A (**A**) and CCND1 (**B**) (Y-axis) in 601 ER+ breast cancer patients from TCGA. The three linear fit curves and 95% C.I. (grey shaded area) demonstrate the interaction effects between FGF2 and FGFR1 expression, where FGFR1 expression levels are grouped into tertiles (high n = 206; low n = 199; med n = 196).


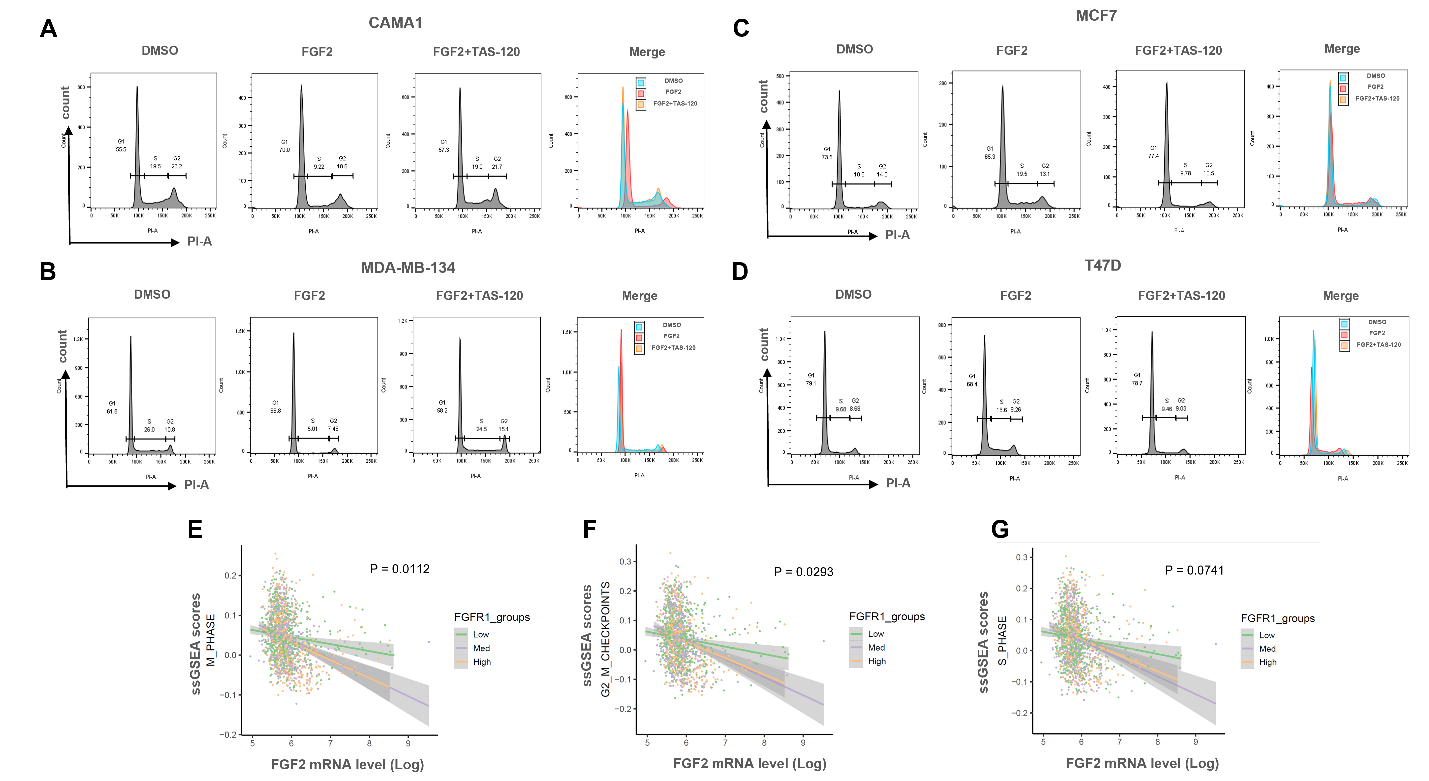
**Figure S9. Reversal of G1 to S transition by TAS-120. A-D.** Detailed FACS analysis plots of cell cycle, where the cell number count is compared with PI-A for four different cell lines: CAMA1 (**A**), MDA-MB-134 (**B**), MCF7 (**C**), and T47D (**D**). The cells were collected from 2D cultures after being exposed to FGF2 (25ng/ml) with or without TAS-120 (1.0 µM) for 24 hours, followed by PI staining-based flow cytometry analysis The combined results of all three treatments (DMSO, FGF2, and FGF2 + TAS-120) are presented on the right in a tri-colored pattern. **E-G.** METABRIC dataset ER+ breast cancer patients data analysis for interactions in the generalized linear model between FGF2 (X-axis): FGFR1 expression and their impact on ssGSEA pathways related to M phase (**C**), G2/M checkpoints (**F**) and S phase (**G**) (Y-axes, n =1445). The three linear fit curves and 95% C.I. (grey shaded area) are shown to indicate the interaction effects between FGF2 and FGFR1 expression, where FGFR1 expression levels were grouped by tertiles (high n =491; low n =477; med n =477).

**
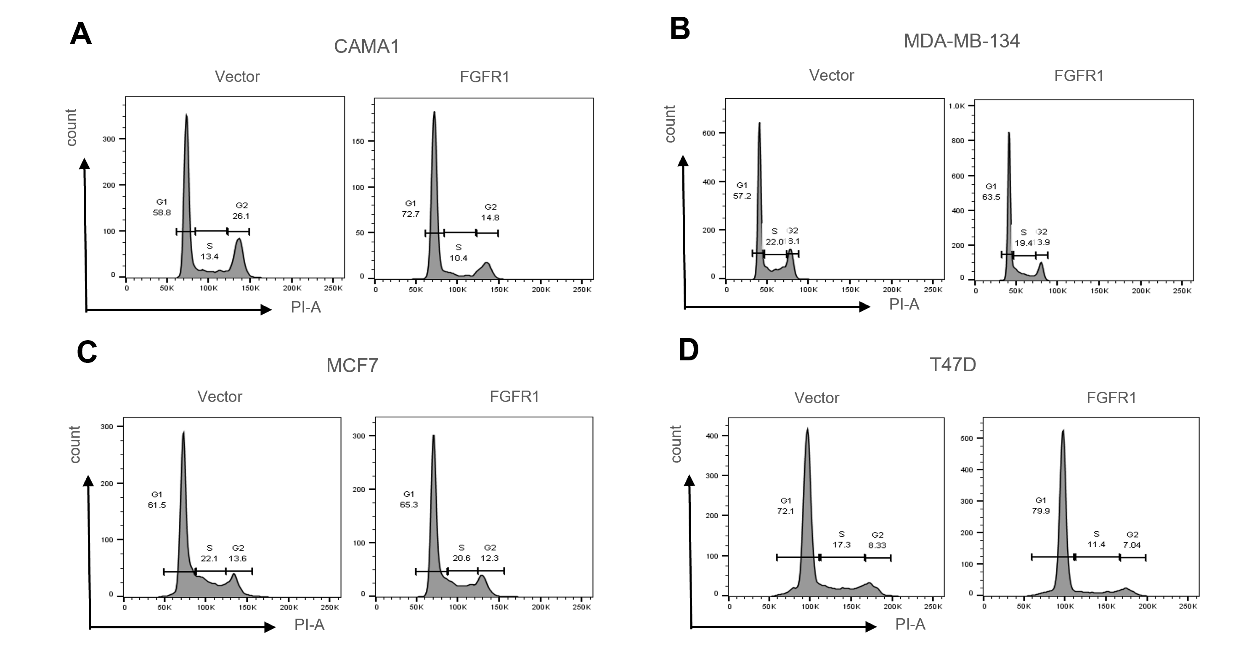
**

F**igure S10. Detailed FACS plots revealed FGFR1 overexpression increased G1 phase in ER+ BC cells. A-D.** Cell cycle FACS analysis plots of cells with transfected FGFR1 or empty vector are indicated by cell number count vs. PI-A for CAMA1 (**A**), MDA-MB-134 (**B**), MCF7 (**C**), and T47D (**D**).


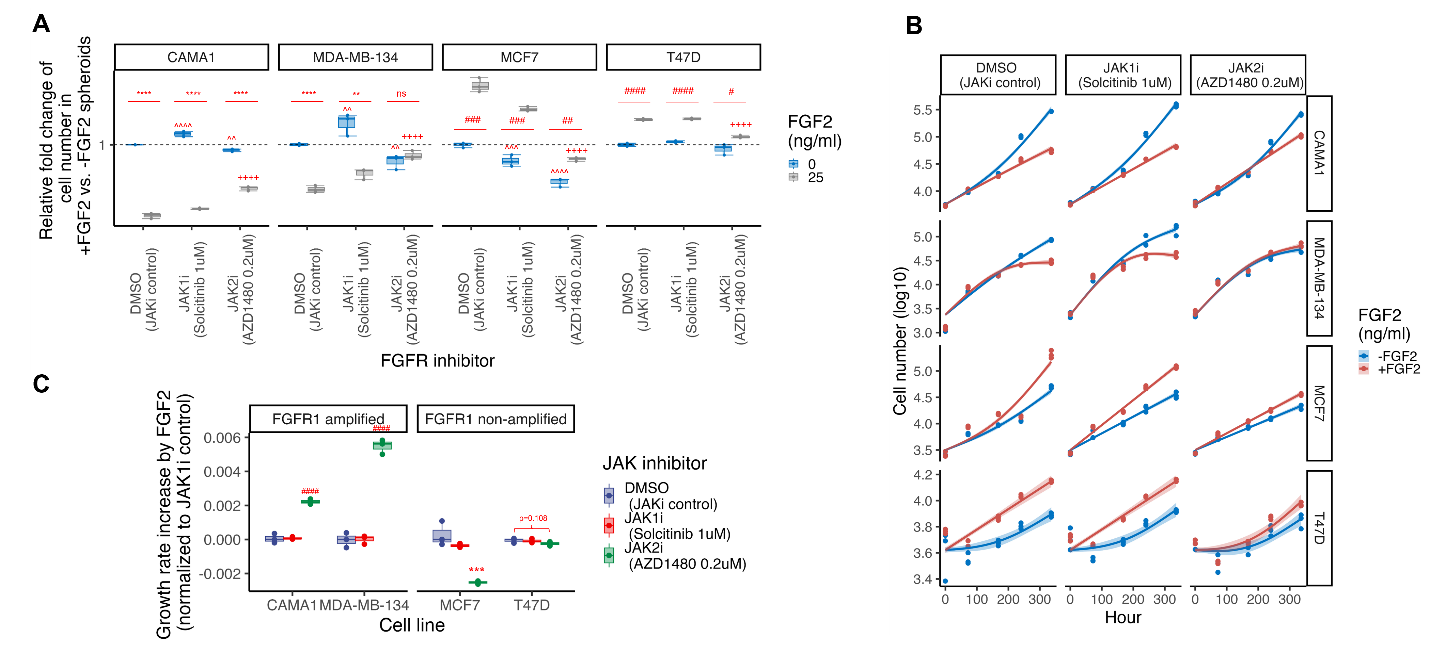


**Figure S11. The JAK2 inhibitor AZD1480 reversed the paradoxical proliferation caused by FGF2. A**. Relative fold change of cell number in +FGF2 vs. -FGF2 spheroids for images in **Fig. 6A** are depicted, with the DMSO control group serving as controls and set to a fold of one. **B**. Time course of cell number change over 14 days 3D culture for two groups of cell lines in **Fig. 6A. C**. Cancer cell growth rate normalized to inhibitor only in each inhibitor group. DMSO treatments serve as the controls in each cell line.


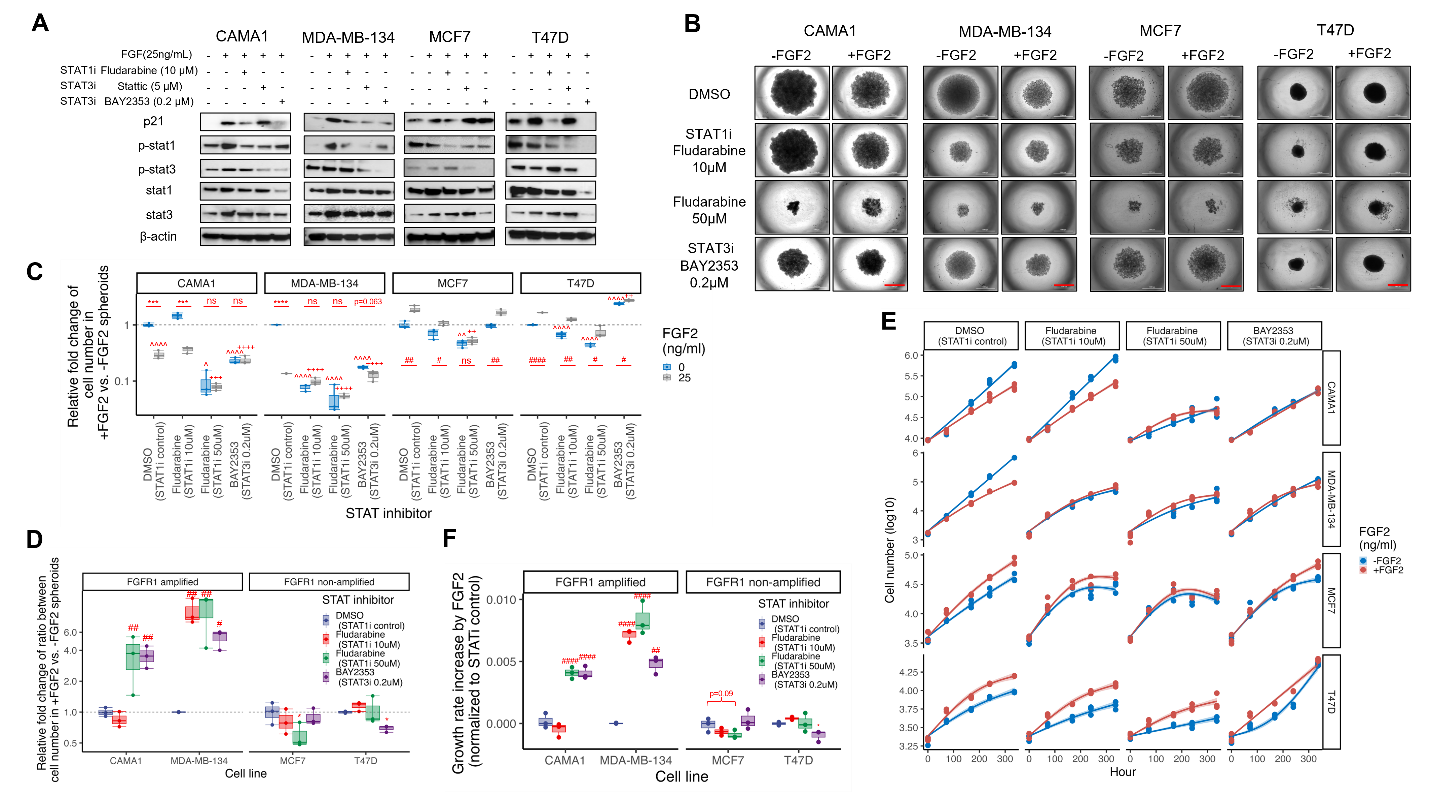
**Figure S12. STAT1 and STAT3 both STAT1 and STAT3 both FGF2 induced mediate proliferation FGF2 induced paradoxical**. **A**. Immunoblotting shows the effects of STAT inhibitors on STAT1/3 activation and p21 levels in 3D cell cultures treated with Fludarabine (10.0 µM), Stattic (5.0 µM) and BAY2353 (0.2 µM) plus FGF2 (25 ng/mL) for 72 hours. **B**. Spheroid images shows the growth effects of Fludarabine (10.0 and 50.0 µM) and BAY2353 (0.2 µM) in FGFR1 amplified cells (CAMA1 and MDA-MB-134) and non-amplified cells (MCF7 and T47D) with and without FGF2 treatment (25 ng/mL) for 14 days. Bar equals 1000 µm. **C**. Relative fold change of ratio between cell numbers in +FGF2 vs. -FGF2 spheroids for images in panel **B**, with the ratios in DMSO treatment group serving as controls and set to a fold of one. **D**. Relative fold change of cell number in +FGF2 vs. -FGF2 spheroids for images in panel **B** are depicted, with the DMSO control group serving as controls and set to a fold of one. **E**. Time course of cell number change over 14 days 3D culture for two groups of cell lines in panel **A.** **F**. Cancer cell growth rate normalized to inhibitor only in each inhibitor dose group. DMSO treatments serve as the controls in each cell line.


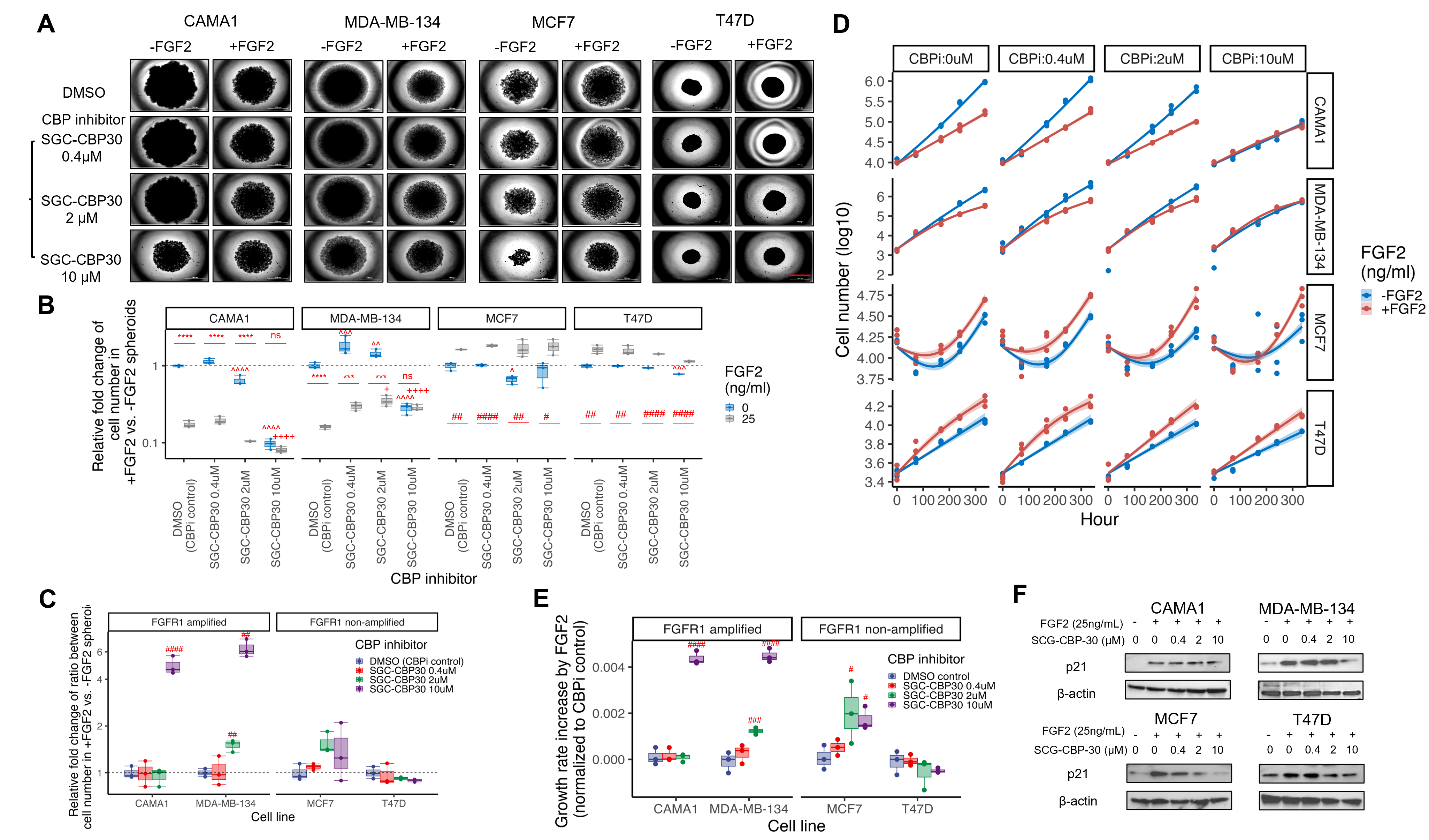


**Figure S13. CBP inhibitor SGC-CBP30 reversed the paradoxical proliferation caused by FGF2.** **A**. Spheroid images show the effects of the CBP inhibitor SGC-CBP30 (0.4, 2.0, and 10.0 µM) on FGFR1 amplified cells (CAMA1 and MDA-MB-134) and non-amplified cells (MCF7 and T47D) with and without FGF2 treatment (25 ng/mL) for 14 days. Bar equals 1000 µm. **B**. Relative fold change of ratio between cell numbers in +FGF2 vs. -FGF2 spheroids for images in panel **A**, with the ratios in DMSO treatment group serving as controls and set to a fold of one. **C**. Relative fold change of cell number in +FGF2 vs. -FGF2 spheroids for images in panel **A** are depicted, with the DMSO control group serving as controls and set to a fold of one. **D**. Time course of cell number change over 14 days 3D culture for two groups of cell lines in panel **A.** **E**. Cancer cell growth rate normalized to inhibitor only in each dose group. DMSO treatments serve as the controls in each cell line.  **F**. Immunoblotting shows the effect of various doses of SGC-CBP30 (1.0, 2.5, and 10 µM) with FGF2 on p21 protein levels in 3D cell cultures after 72 hours of treatment with FGF2 (25 ng/mL).


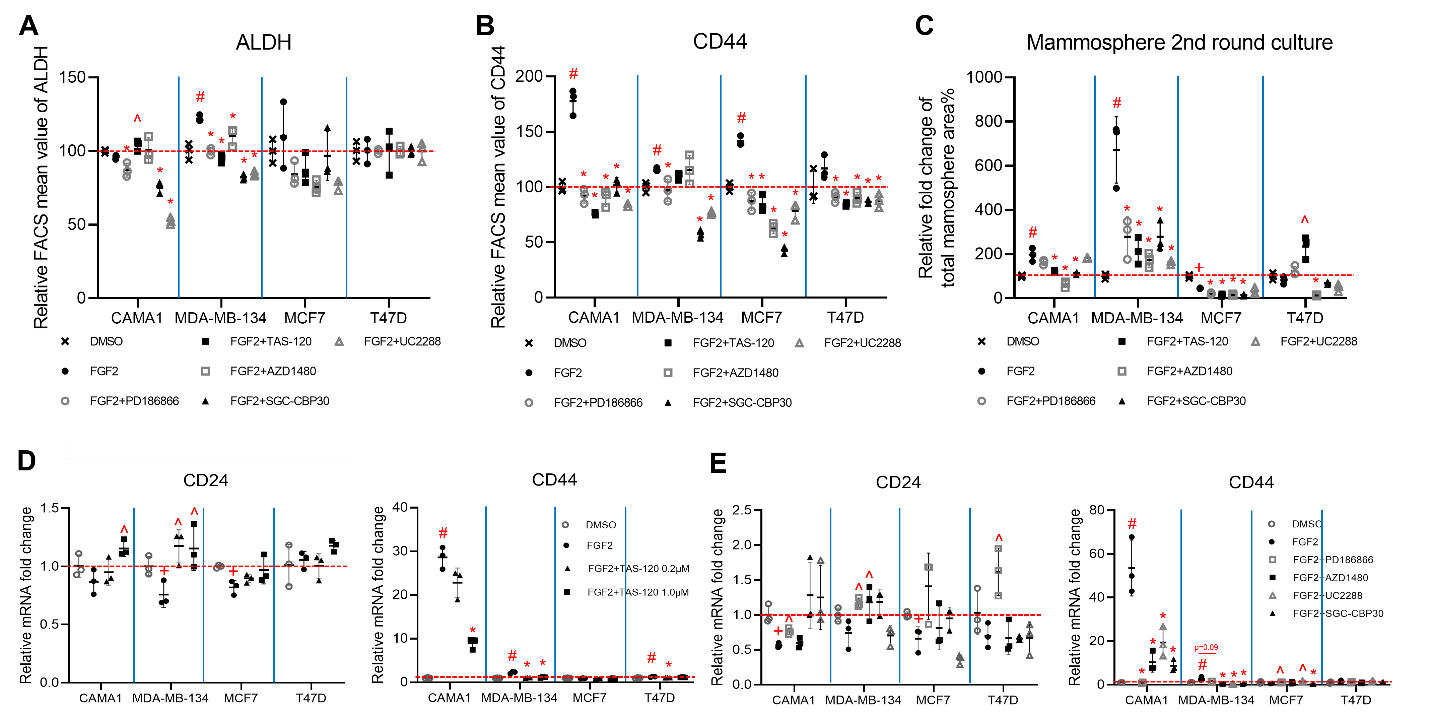


**Figure S14. FGF2 increases cancer stemness through JAK-STAT pathway.** **A-C.** Relative FACS mean value of ALDH (**A**), CD44 (**B**) and relative percentage change of total mammosphere area for the 2^nd^ round culture (**C**) are shown in four ER+ cell 3D cultures incubated with FGF2 (25ng/mL) plus PD166866 (1.0µM), TAS-120 (1.0µM), AZD1480 (0.2µM), SGC-CBP30 (10µM) and UC2288 (1.0µM). Cells from spheroids were incubated with FGF2 plus inhibitors for 72 hours before ALDEFLUOR/CD44 staining. Mammosphere colonies harvested from 1^st^ round culture were dissociated and counted, live cells were replated for 2^nd^ round of mammosphere culture under the same condition for another 7 days. **D**. In ER+ cell 3D cultures, the mRNA expression levels of stemness genes CD24 and CD44 were assessed after a 72-hour incubation with FGF2 (25 ng/mL) and TAS-120 at different doses (0.2 and 1.0 µM). **E**. The mRNA expression levels of stemness genes CD24 and CD44 were measured in ER+ cell 3D cultures following a 72-hour incubation with FGF2 (25 ng/mL) combined with PD186866 (1.0 µM), AZD1480 (0.2 µM), UC2288 (1.0 µM), and SGC-CBP30 (10 µM). The mRNA expression levels were normalized to RPLP0 levels, with control treatments using DMSO only set as a fold of one.


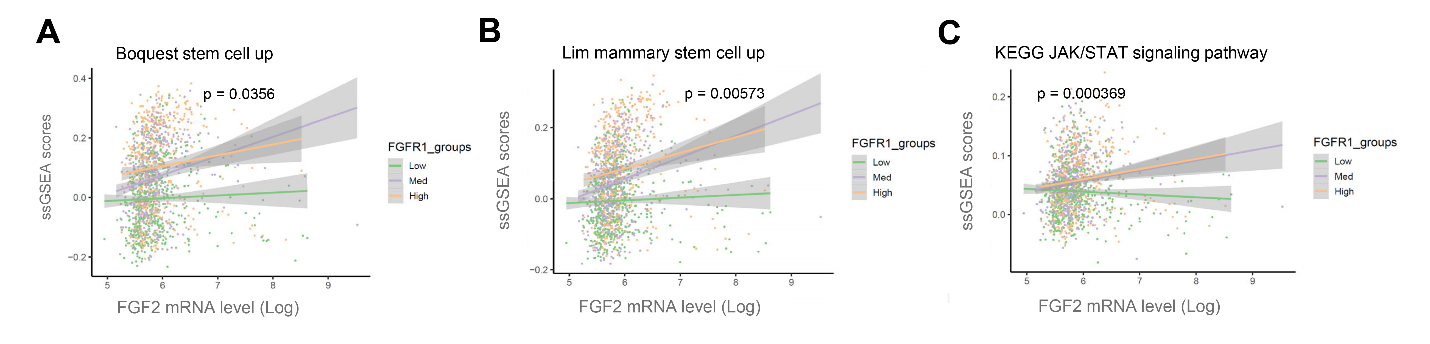
**Figure S15. METABRIC dataset analysis for the correlation between FGF2 and FGFR1 expression and its impact on stemness pathways.** The analysis utilized a linear model like **Fig. 7 C-F** to examine the interaction between FGF2 (X-axis) and FGFR1 expression (Y-axis) and its effect on stemness (**A-B**) and JAK-STAT (**C**) pathways using ER+ breast cancer patients from the METABRIC dataset (n=1445). Three linear fit curves with 95% C.I. (grey shaded area) are presented to indicate the interaction between FGF2 and FGFR1 expression. The FGFR1 expression is divided into three groups based on tertiles: high (n=491), low (n=477), and medium (n=477).

# Uncropped gel and blot images


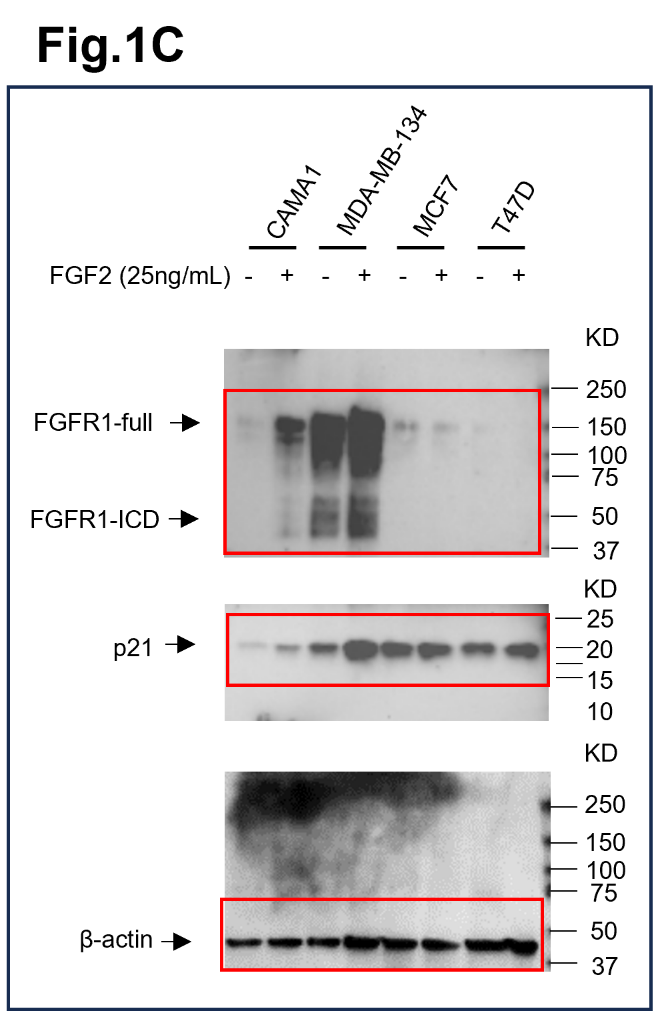


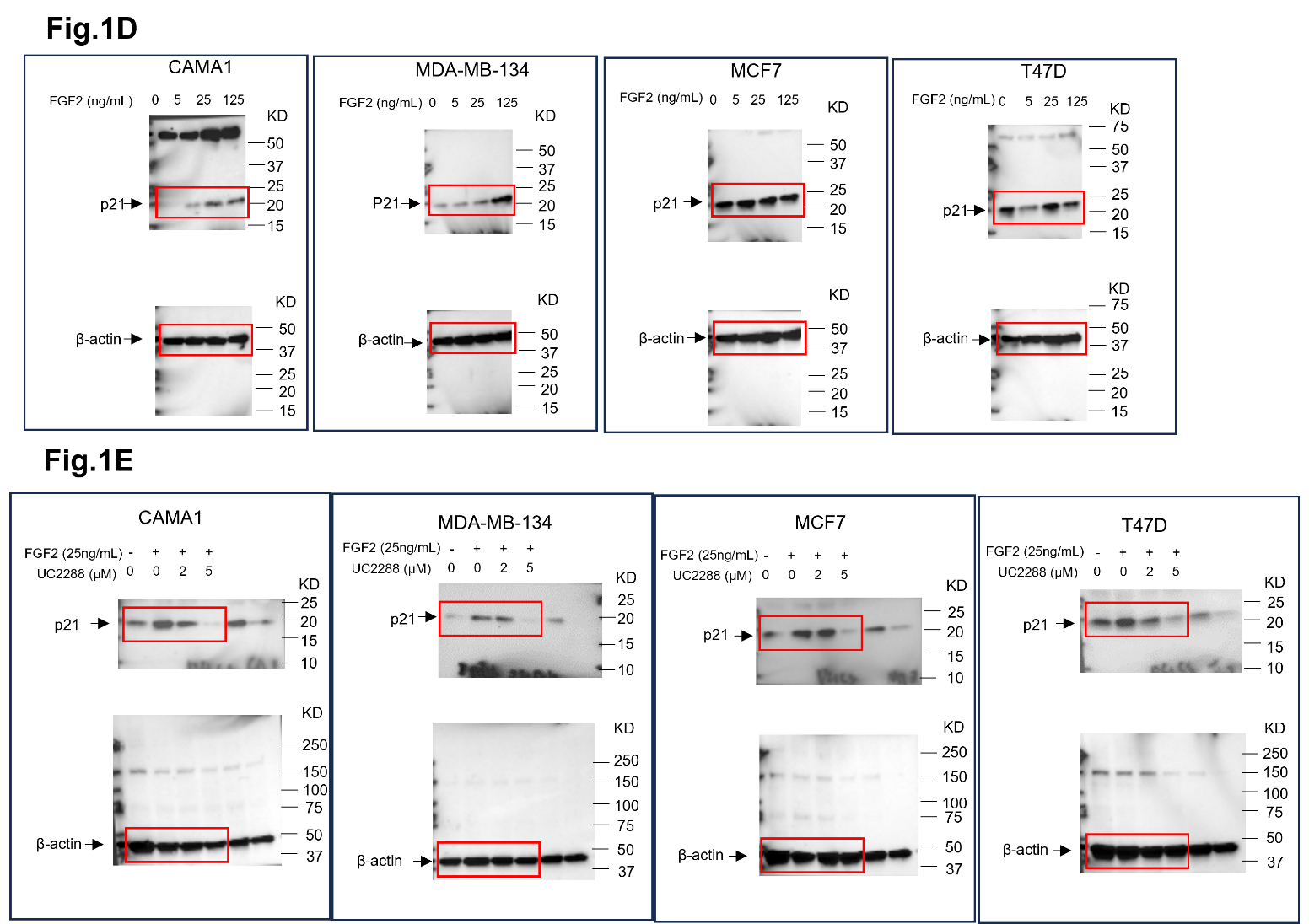


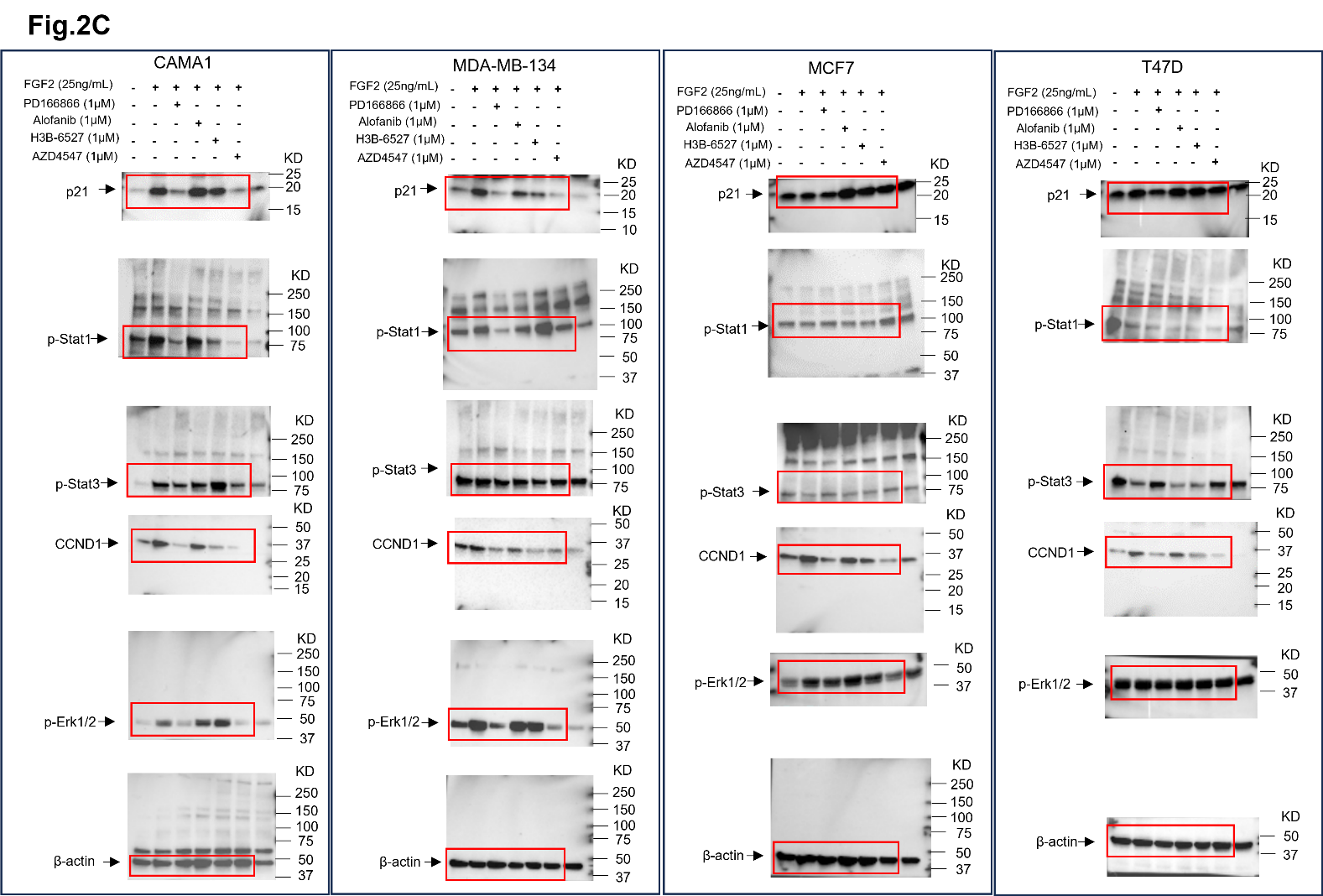


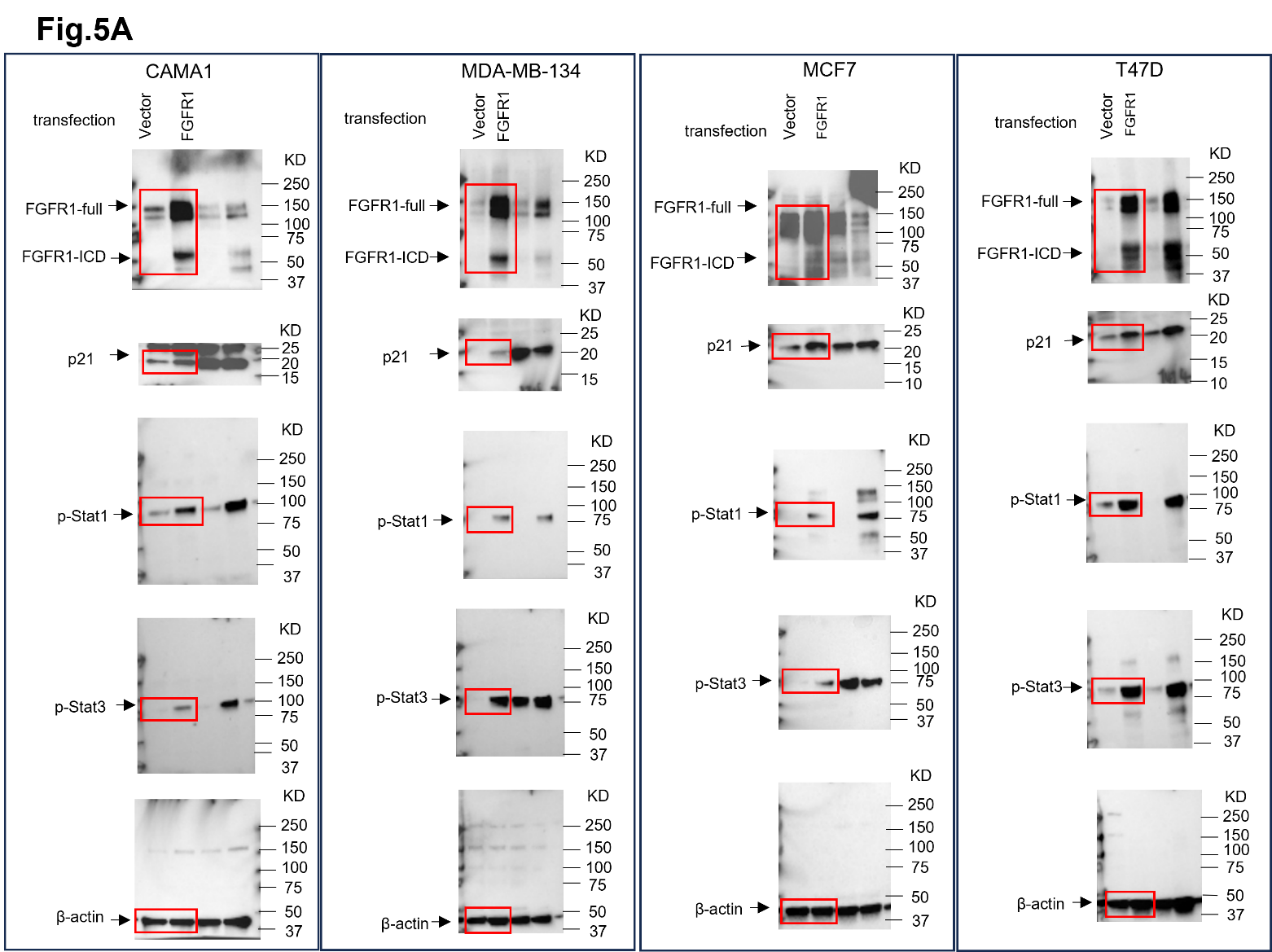


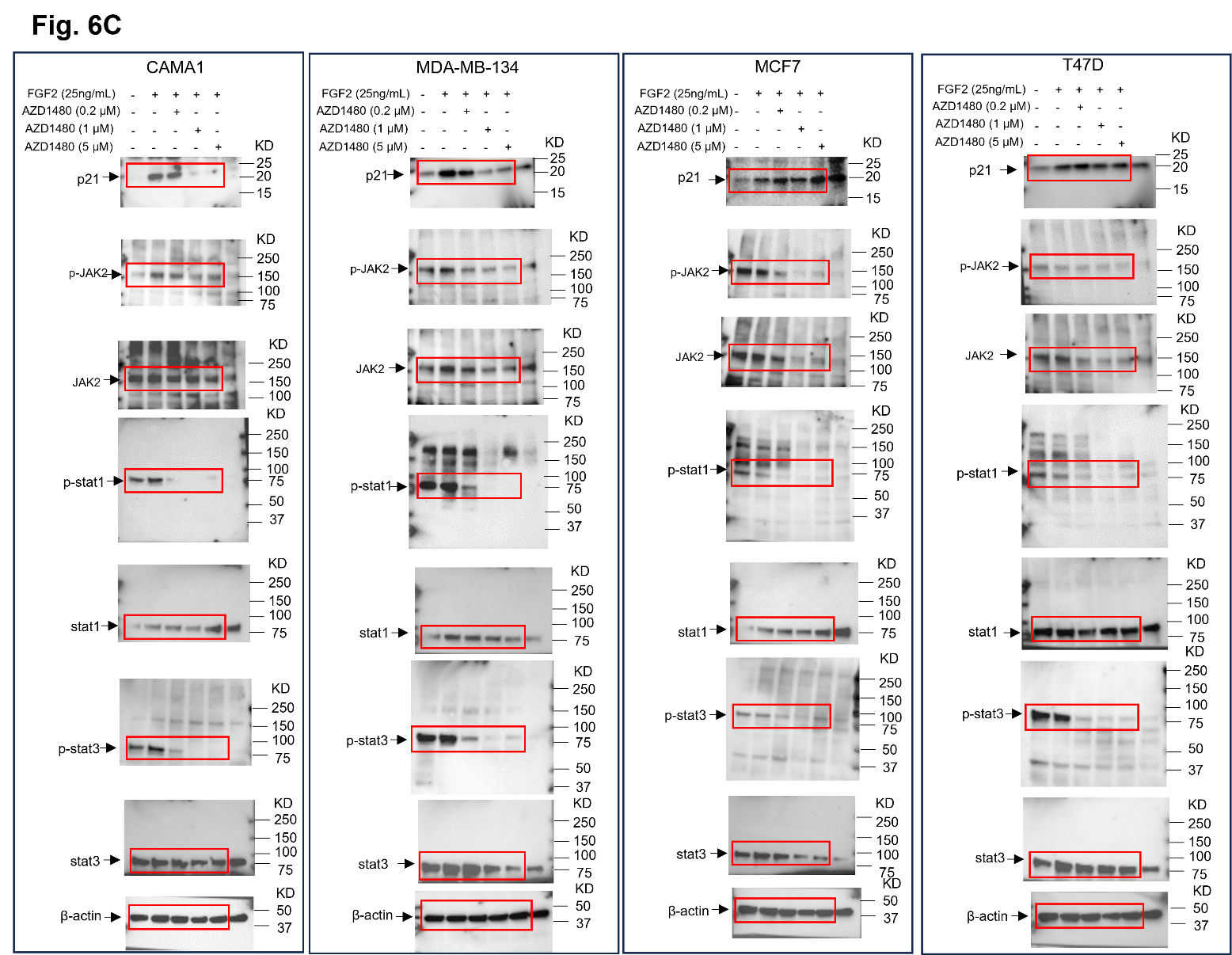


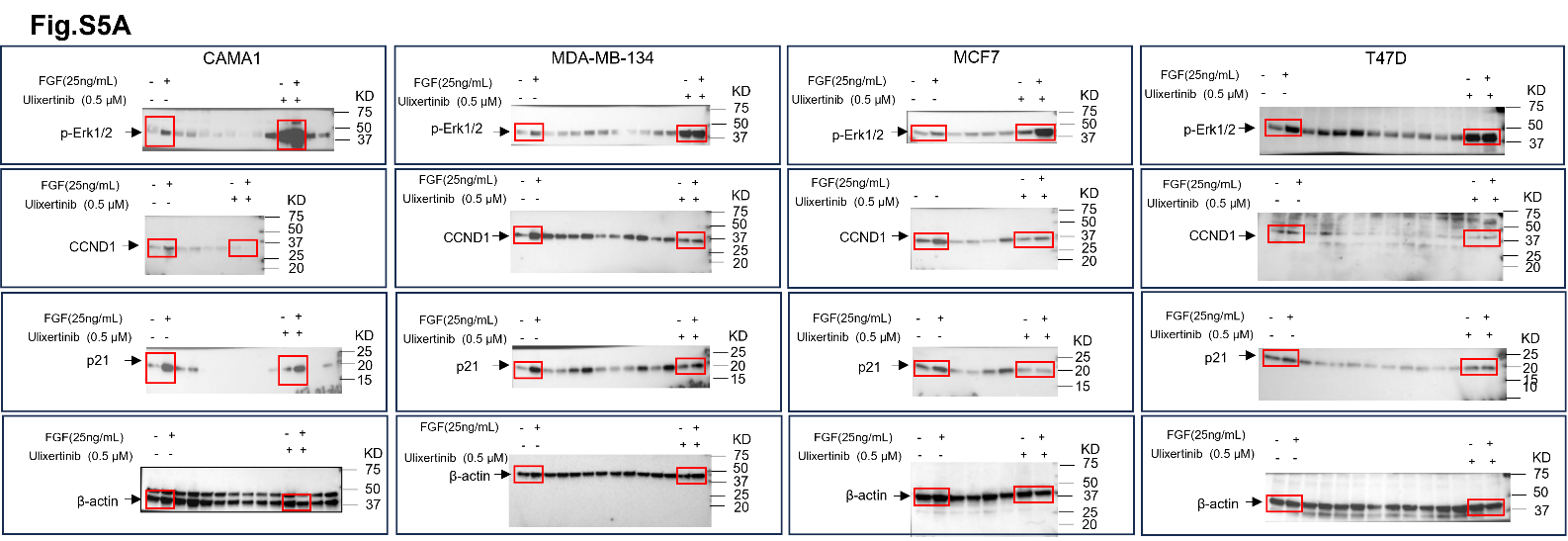


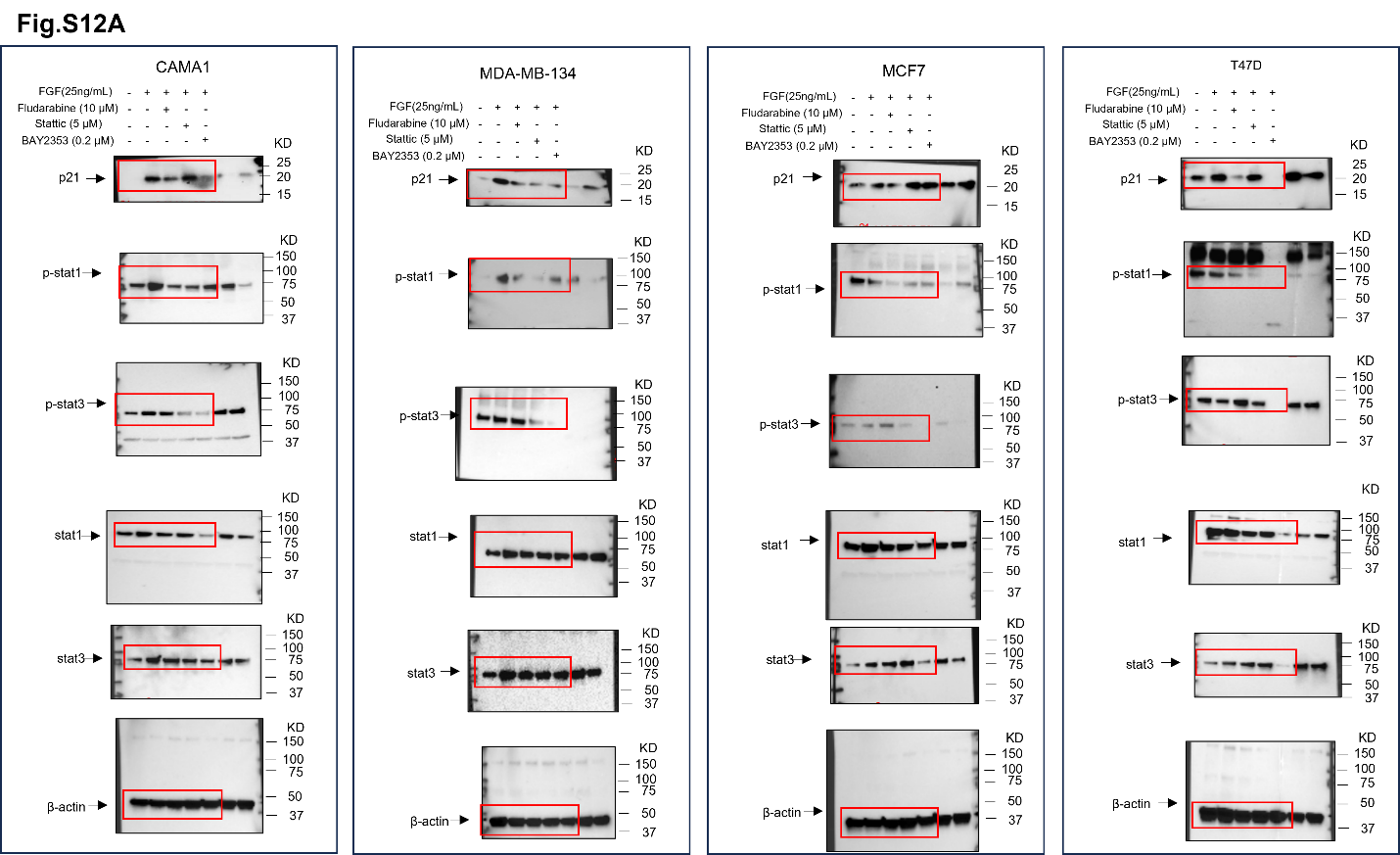


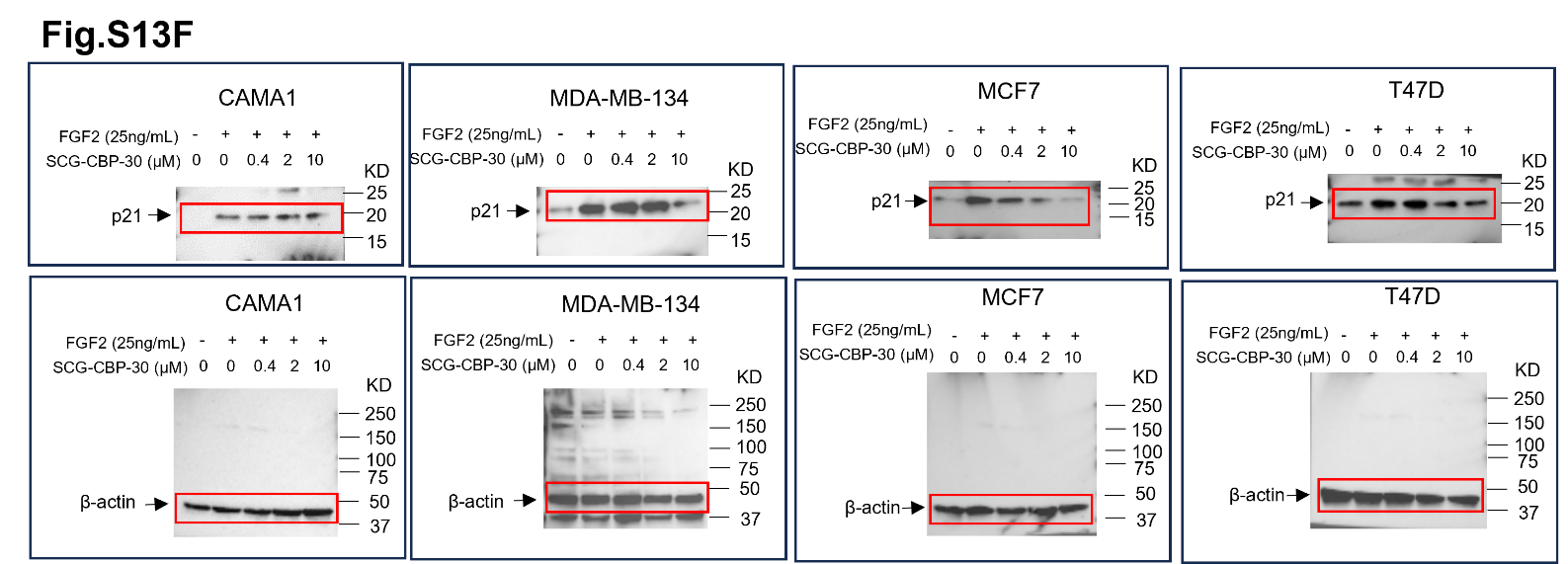

Supplement: Supplementary file 1 — Supplementary Material 1 [file 13058_2024_1808_MOESM1_ESM.docx]
